# Younger adult brain utilizes interhemispheric strategy via ipsilateral dorsal premotor cortex for fine control of dexterous finger movements, unlike the aging brain

**DOI:** 10.3389/fnagi.2025.1501011

**Published:** 2025-07-21

**Authors:** Gen Miura, Tomoyo Morita, Jihoon Park, Eiichi Naito

**Affiliations:** ^1^Center for Information and Neural Networks (CiNet), Advanced ICT Research Institute, National Institute of Information and Communications Technology (NICT), Osaka, Japan; ^2^Graduate School of Frontier Biosciences, The University of Osaka, Osaka, Japan; ^3^Symbiotic Intelligent Systems Research Center, Institute for Open and Transdisciplinary Research Initiatives, The University of Osaka, Osaka, Japan

**Keywords:** ipsilateral premotor cortex, ipsilateral sensorimotor cortices, dexterous motor task, fine control, coordination of individual fingers, aging, functional MRI, functional connectivity

## Abstract

**Introduction:**

This study investigated how healthy, right-handed younger adults utilize the typically suppressed ipsilateral sensorimotor cortices [particularly, the dorsal premotor cortex (PMd), primary motor cortex (M1), primary somatosensory cortex (S1), and superior parietal cortex of Area 2] to perform a dexterous finger motor task and its age-related changes.

**Methods:**

Functional magnetic resonance imaging was used to measure brain activity in healthy, right-handed younger and older adults during a simple button-press task with the right index finger and a dexterous stick-rotation task involving fine control and coordination of individual right finger movements. The individual performance capacity in stick rotation (the personal trait of finger dexterity) was assessed outside the scanner. The sensorimotor cortices (PMd, M1, S1, and Area 2) in each hemisphere were defined as regions-of-interest (ROIs), and contrast analysis, interparticipant correlation analysis with performance capacity, and interhemispheric functional connectivity analysis were performed.

**Results:**

In the younger group, all ipsilateral sensorimotor cortices were deactivated during the button-press task, whereas during the stick-rotation task, the PMd, S1, and Area 2 were activated, and the ipsilateral M1 remained deactivated. The ipsilateral PMd, S1, and Area 2 activity was correlated with performance capacity. During the stick-rotation task, the anterior ipsilateral PMd consistently enhanced interhemispheric functional coupling with all contralateral sensorimotor cortices. In contrast, in the older group, ipsilateral sensorimotor deactivation was not observed during the button-press task, and all ipsilateral cortices were activated during the stick-rotation task; however, none of the activity was correlated with performance capacity. In addition, functional connectivity within the contralateral sensorimotor cortices (rather than interhemispheric connectivity) increased during the stick-rotation task.

**Conclusion:**

Our findings indicate that ipsilateral sensorimotor activity during the current dexterous task reflects different physiological mechanisms between younger and older adults. When performing the task, younger adults recruited the ipsilateral PMd, S1, and Area 2 by disinhibiting their interhemispheric inhibition to complement for their clumsiness; the ipsilateral PMd appeared important for the interhemispheric interaction, whereas the ipsilateral sensorimotor activity in older adults did not appear to represent proactive interhemispheric interaction to overcome clumsiness.

## 1 Introduction

The human sensorimotor system is highly plastic. For example, when the brain or spinal cord is injured, the brain often recruits typically underused cortical sensorimotor areas [the dorsal premotor cortex (PMd), the primary motor cortex (M1), the primary somatosensory cortex (S1), and the superior parietal cortex of Area 2] for ipsilateral hand/finger movements (Grefkes and Fink, 2020; Hoy et al., 2004; Jang et al., 2004; Lotze et al., 2006; Ward et al., 2008). In stroke patients, jamming transcranial magnetic stimulation (TMS) to the ipsilateral sensorimotor cortices disrupts finger movement, especially the TMS to the PMd disrupts the movement more than when it is applied to the M1 and the superior parietal lobule (Lotze et al., 2006). It is also shown in non-human primates that the ipsilateral PMd is an important node in the recovery of grasping function after the spinal cord injury (Chao et al., 2019). These findings suggest that the ipsilateral sensorimotor cortices are capable of complementing sensorimotor control of finger movement, especially the ipsilateral PMd plays a particularly important role (Fracasso et al., 2022; Johansen-Berg et al., 2002).

In healthy younger adults, the ipsilateral sensorimotor cortices are typically deactivated during simple sensory or motor tasks (Allison et al., 2000; Hayashi et al., 2008; Marchand et al., 2007; Morita et al., 2019, 2021; Mullinger et al., 2014; Naito et al., 2021; Newton et al., 2005; Stefanovic et al., 2004). This deactivation is thought to result from interhemispheric inhibition from the contralateral cortices (Ferbert et al., 1992; Kobayashi et al., 2003; Mullinger et al., 2014; Talelli et al., 2008). In contrast, as observed in the aforementioned clinical cases, ipsilateral sensorimotor activation has been consistently reported during demanding dexterous hand and finger motor tasks. This activity is thought to complement motor performance (Loibl et al., 2011; Nambu et al., 2015; Uehara et al., 2012, 2019; Verstynen et al., 2005), possibly by disinhibiting interhemispheric inhibition. If this interpretation holds true, the ipsilateral sensorimotor cortices are deactivated during simple finger movements in younger adults. In contrast, it may contribute to motor performance during fine finger movements via increased functional coupling with the contralateral cortices. Based on the clinical evidence following brain or spinal cord injury in both humans (Lotze et al., 2006; Fracasso et al., 2022; Johansen-Berg et al., 2002) and non-human primates (Chao et al., 2019) demonstrating that the ipsilateral sensorimotor cortices—particularly the PMd—plays a crucial compensatory role in the sensorimotor control of finger movements, we hypothesize that the ipsilateral PMd serves as a core region for complementary function.

By contrast, the ipsilateral sensorimotor cortices in older adults can exhibit activity during both simple unilateral motor tasks (Hutchinson et al., 2002; Riecker et al., 2006; Morita et al., 2021) and kinesthetic stimulation without overt movement (Naito et al., 2021), unlike in younger adults. Such activation is thought to result from age-related decline in interhemispheric inhibition between the bilateral sensorimotor cortices (Hutchinson et al., 2002; Mattay et al., 2002; Morita et al., 2021; Naito et al., 2021; Riecker et al., 2006; Talelli et al., 2008). Additionally, activation of the ipsilateral sensorimotor cortices has been reported in older adults during the performance of complex, dexterous tasks (Loibl et al., 2011). At first glance, this activation may appear similar to that observed in younger adults; however, the underlying neural mechanisms and functional significance may differ between the two age groups. Of particular importance is whether the ipsilateral activation observed in older adults serves a complementary role, as would be supposed in younger adults. This question arises in part because, in the cognitive domain, older adults who exhibit greater bilateral activation of the prefrontal cortices tend to perform better on memory tasks—a phenomenon widely known as hemispheric asymmetry reduction in older adults (HAROLD; Cabeza, 2002; Cabeza et al., 2018). If the HAROLD model also applies to sensorimotor domains, the ipsilateral sensorimotor cortices in older adults may serve a complementary role in coordination with the contralateral cortices. Conversely, if this is not the case, interhemispheric functional coupling may not be enhanced, and the ipsilateral activation itself may be unrelated to complementary function.

**FIGURE 1:**
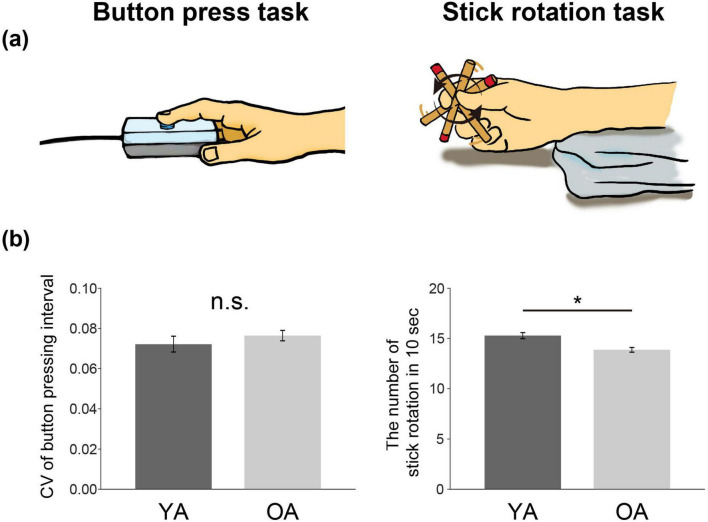
Motor tasks **(a)** and performance **(b)**. **(a)** Button-press task using the right index finger to evaluate ipsilateral sensorimotor suppression (left panel). Stick-rotation task using the right thumb, index, and middle fingers to evaluate the complementary roles of ipsilateral sensorimotor areas (right panel). **(b)** Left panel: Performance in the button-press task measured inside the scanner. Right panel: Performance capacity of stick rotation (i.e., maximum number of stick rotations in 10 s measured outside the scanner). Dark gray bars indicate data from the YA group and light gray bars indicate data from the OA group. Error bars indicate standard error of the mean (SEM) across participants. *indicates *p* < 0.05. CV, coefficient of variation; YA, younger adult; OA, older adult; n.s., not significant.

To address these questions, the present study included two finger motor tasks. As a simple motor task, we developed a simple button-press task using the right index finger (Figure 1a, left panel). As a dexterous motor task, we developed a stick-rotation task requiring fine control and coordination of three fingers in the right hand (Figure 1a, right panel). The former was used to assess ipsilateral sensorimotor suppression, whereas the latter was used to examine the role of the ipsilateral sensorimotor activity during the dexterous task (Figure 1a, right panel). We measured brain activity using functional magnetic resonance imaging (fMRI) when healthy younger and older adults performed these two tasks at a constant rhythm.

In this study, we set regions-of-interest (ROIs) in each hemisphere’s sensorimotor cortices (PMd, M1, S1, and Area 2). First, we carefully evaluated the activation and deactivation of each task in each group. Second, as an index of the personal trait of finger dexterity, the individual performance capacity (level) in stick rotation was evaluated by measuring the maximum number of stick rotations outside the scanner, and we performed an interparticipant correlation analysis to examine whether the ipsilateral sensorimotor activity during the fixed-paced stick-rotation task increases in relation to the performance capacity. Finally, we examined brain regions in the sensorimotor ROIs that exhibited increased functional coupling with their respective contralateral seed regions (PMd, M1, S1, and Area 2) during the stick-rotation task compared with that during the button-press task.

## 2 Materials and methods

### 2.1 Participants

This was an open-label study, in which both researchers and participants know which experiments are being administered. Healthy right-handed younger adults (YA group: 22 men, 9 women: mean age, 22.1 ± 1.8 years) and older adults (OA group: 31 men, 17 women: mean age, 71.1 ± 4.3 years) participated in this study. Appropriate sample size for an fMRI study is still under debate. Thus, we determined the sample size for younger adults, with reference to a recent review reporting the median sample size in fMRI studies as 24 participants in 2018 along with a yearly increase of approximately 0.74 participants (Szucs and Ioannidis, 2020). Previous studies have suggested that older adults generally have greater inter-individual variability and an aging study usually requires larger sample sizes to achieve sufficient statistical power (Samanez-Larkin and D’Esposito, 2008). Accordingly, we recruited approximately 1.5 times as many older participants as younger participants.

The cognitive status of older participants was assessed using the Mini-Mental State Examination. All participants scored higher than the cutoff score of 24 (Lopez et al., 2005). The handedness of the participants was confirmed using the Edinburgh Handedness Inventory (Oldfield, 1971). We also verified the absence of a history of neurological, psychiatric, or motor disorders based on self-reports.

The study protocol was approved by the Ethics Committee of the National Institute of Information and Communications Technology and the MRI Safety Committee of the Center for Information and Neural Networks (CiNet; no. 2003260010). All participants were informed about the study before the experiment, and written informed consent was obtained. The study was conducted according to the principles and guidelines of the Declaration of Helsinki (1975).

### 2.2 Motor tasks

We placed the participants in a supine position inside an MRI scanner, where they performed two motor tasks: a button-press task and a stick-rotation task. To standardize the number of movements across participants during brain activity measurements, we instructed them to perform movements according to a constant periodic sound (see below). We also instructed the participants to close their eyes, relax their bodies, avoid unnecessary movements, and focus on the assigned tasks.

(1) *Button-press task*: The participants were asked to press a magnetic resonance–compatible button (Current Design Inc., Philadelphia, PA) with their right index finger in synchronization with a computer-generated sound at a frequency of 1 Hz (Figure 1a, left panel). Based on previous reports that the ipsilateral sensorimotor cortices (PMd, M1, S1, and Area 2) in younger adults are typically suppressed during simple motor and kinesthetic tasks (Riecker et al., 2006; Morita et al., 2021; Naito et al., 2021), we adopted this task to determine the suppression of ipsilateral sensorimotor activity. In the present study, we use the term ‘ipsilateral’ to refer to the right hemisphere (i.e., the hemisphere on the same side as the moving hand) and ‘contralateral’ to refer to the left hemisphere (i.e., the hemisphere on the opposite side). Throughout each fMRI run, the participants maintained their right index fingers on the button, repetitively pressing the button without releasing the finger from it. To evaluate the performance during the button-press task in both YA and OA groups, we used the coefficient of variation (CV) of the button pressing interval recorded when performing the task during scanning (Figure 1b, left panel). In the analysis, we excluded data from one younger participant because his data exceeded ± 2 standard deviations (SDs) of the mean CV of the YA group. The mean CV across participants was calculated in each group, and between-group differences were assessed using Welch’s *t*-test without the equal-variance assumption (Figure 1b, left panel).

(2) *Stick-rotation task*: The participants were asked to rotate a 9.8-cm, 21-g wooden stick 180°counterclockwise with their right hand (using thumb, index, and middle finger) in synchronization with a computer-generated sound at a frequency of 0.8 Hz (Figure 1a, right panel). We selected this task as a dexterous motor task because it requires fine control and coordination of individual finger movements of the right hand; we expected an increase in the ipsilateral sensorimotor activity based on previous reports (Loibl et al., 2011; Nambu et al., 2015; Uehara et al., 2012, 2019; Verstynen et al., 2005). Preliminary experiments revealed that some older participants had difficulty rotating the stick at 1 Hz. Therefore, we selected a frequency of 0.8 Hz, which allowed the participants to perform the task successfully. Throughout each fMRI run, we visually confirmed that participants performed the 0.8-Hz stick rotation.

The participants performed each task in two sessions (a total of four sessions). One session comprised five task epochs of 15 s, each alternating with five rest epochs (baseline state) of 15 s, starting with the rest epoch. Additionally, we provided an extra 10 s before the first rest epoch for magnetization stabilization. Thus, one session lasted 160 s. During the rest epochs, the participants received auditory stimuli at 1 Hz (button-press task) or 0.8 Hz (stick-rotation task) but did not move their fingers. As the duration of eye closure may affect brain activity (Merabet et al., 2007; Weisser et al., 2005), the participants were instructed to close their eyes before each session. Half of the participants in each group were randomly assigned to perform the button-press task first, whereas the other half performed the stick-rotation task first. During each fMRI run, the participants received computer-generated auditory instructions (“3, 2, 1, start” and “stop”) through a magnetic resonance–compatible headphone to inform them of the beginning and end of a task epoch. The timing of each participant’s sounds and button pressing was also recorded.

(3) *Evaluation of individual performance capacity in stick rotation*: In the scanner, we measured brain activity while participants performed the stick-rotation task at a constant rhythm (0.8 Hz). To evaluate each participant’s individual performance capacity (level) in stick rotation, we also assessed their maximum performance on the stick-rotation task outside the MRI scanner and used this data in the subsequent correlation analysis. Outside the scanner, participants were seated comfortably in a chair with their hands placed on a table and were instructed to perform the stick-rotation task as quickly as possible. The goal was to capture their best performance, thereby providing a reliable measure of individual capacity. We did not provide specific instructions regarding visual input. When asked about visual use, we told participants they could keep their eyes open or closed, depending on what felt most comfortable. All participants kept their eyes open, except for one older adult. However, approximately 20% of participants in each group, although keeping their eyes open, did not visually track their finger movements. This proportion was comparable between the two groups. Notably, body posture and visual conditions during this out-of-scanner assessment differed from those during the constant-paced stick-rotation task performed in the scanner. For the performance measurement, participants were instructed to rotate the stick as many times as possible within 10 s. We quantified performance by counting the number of 180° rotations completed. Each participant completed three trials of this task, and we used the average number of rotations as the individual performance capacity in the stick-rotation task (Figure 1b, right panel).

In the analysis, we excluded a younger participant (not the one excluded in the button-press task) because his data exceeded ± 2 SD of the mean performance capacity in stick rotation in the YA group. The mean performance capacity of stick rotation across participants was calculated in each group, and between-group differences were evaluated using Welch’s *t*-test (Figure 1b, right panel).

### 2.3 Acquisition of fMRI

We acquired functional images using T2*-weighted gradient echo-planar imaging (EPI) sequences on a 3.0-Tesla MRI scanner (Trio Tim; Siemens, Germany) equipped with a 32-channel array coil. Each volume comprised 44 slices (slice thickness, 3.0 mm; inter-slice thickness, 0.5 mm) acquired in ascending order, covering the entire brain. We used a time interval of 2,500 ms between successive acquisitions from the same slice, an echo time (TE) of 30 ms, and a flip angle (FA) of 80°. We used a field of view (FOV) of 192 × 192 mm^2^ and a matrix of 64 × 64 pixels. The dimensions of the voxel on the x-, y-, and z-axes were 3 × 3 × 3.5 mm^3^, respectively. For each experimental run, 65 volumes were collected. As an anatomical reference, we acquired a T1-weighted magnetization-prepared rapid gradient echo image using the same scanner. The imaging parameters were as follows: repetition time = 1,900 ms, TE = 2.48 ms, FA = 9°, FOV = 256 × 256 mm^2^, matrix size = 256 × 256 pixels, slice thickness = 1.0 mm, voxel size = 1 × 1 × 1 mm^3^, and 208 contiguous transverse slices.

### 2.4 Functional image analysis

#### 2.4.1 Preprocessing

To eliminate the influence of unsteady magnetization during tasks, we excluded the first four volumes (10 s) of the EPI images in each run from the analysis. We analyzed the acquired imaging data using SPM 12 (default setting: Wellcome Center for Human Neuroimaging, London, United Kingdom) running on MATLAB R2017a (MathWorks, Sherborn, MA, United States).

The EPI images were realigned to correct for head motion. Time series data of the head position during the fMRI experiment were obtained by a rigid body transformation (linear transformation) using the least squares method for six realign parameters (translation along the x-, y-, and z-axes and the rotational displacements of pitch, raw, and roll). Thereafter, head movements were evaluated by the framewise displacement (FD) values based on the six parameters (Power et al., 2012). To inspect the FD values through all frames of an entire experimental run, for each participant, we counted the number of frames with an FD of > 0.9 mm, as reported in a previous study (Siegel et al., 2014). We excluded two older participants with FD values of > 0.9 mm in > 5% of the total volumes from subsequent behavioral and imaging analyses. A non-linear transformation was also performed to correct distortions in the functional brain images due to inhomogeneities in the magnetic field caused by the participant’s head movement (unwarp). Next, the T1-weighted structural image was coregistered with the mean image of each participant’s realigned and unwarped EPI images. The individual coregistered T1-weighted structural image was spatially normalized to the standard stereotactic Montreal Neurological Institute (MNI) space (Evans et al., 1994). Applying the parameter estimated in this process, individual realigned and unwarped images were normalized to the MNI space with a 2-mm isotropic voxel size using the SPM12 normalization algorithm. Finally, normalized images were filtered along the x-, y-, and z-axes using a Gaussian kernel with a full-width at half-maximum (FWHM) of 4 mm.

#### 2.4.2 Single-participant analysis

After preprocessing, we first explored task-related activations and deactivations in each participant using a general linear model (Friston et al., 1995; Worsley and Friston, 1995). For the first-level analysis, we prepared a design matrix for each participant. The design matrix contained a boxcar function convolved with a canonical hemodynamic response function (HRF) for each task epoch. In addition, we included six realignment parameters in the design matrix as regressors to correct for residual motion-related noise after realignment. We created contrast images showing activation (task > rest) and deactivation (rest > task) in the button-press and stick-rotation tasks for each participant. Furthermore, we did not perform global mean scaling to avoid inducing a type I error when assessing negative blood oxygenation-level dependent (BOLD) responses (Aguirre et al., 1998).

#### 2.4.3 ROIs

**FIGURE 2:**
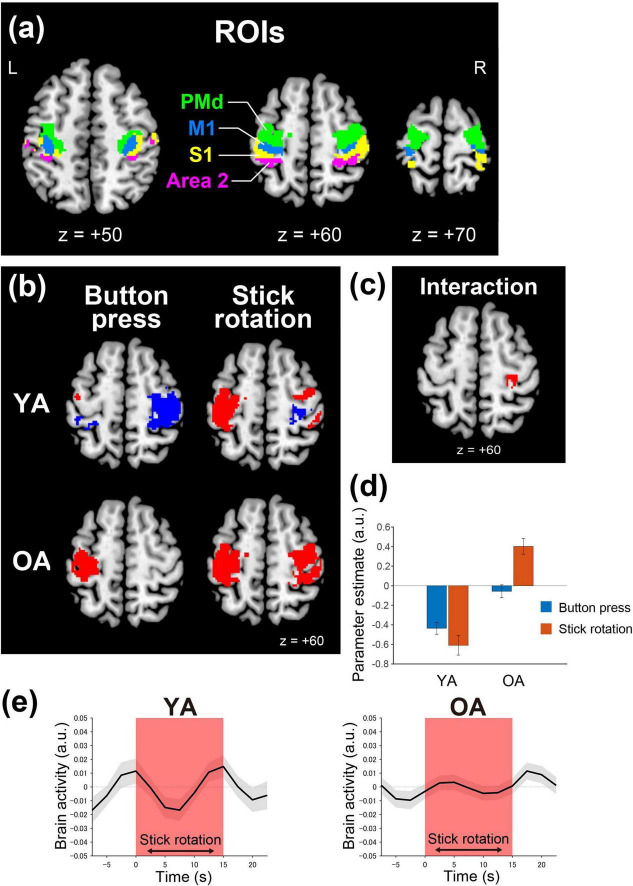
ROIs in hand/finger sections of the bilateral PMd, M1, S1, and Area 2 **(a)**, and contrast and temporal profile analyses **(b–e)**. **(a)** Bilateral sensorimotor ROIs [PMd (green), M1 (blue), S1 (yellow), and Area 2 (magenta)] superimposed on transverse sections (*z* = + 50, 60, and 70) of the MNI standard brain. **(b)** Brain activation (red) and deactivation (blue) during the button-press task (left) and the stick-rotation task (right) in the YA (top row) and OA (bottom row) groups, superimposed on a transverse section (*z* =+60) of the MNI standard brain. **(c)** Ipsilateral M1 (red) showing a significant interaction between task and group ([stick rotation>button press in OA]>[stick rotation>button press in YA]), superimposed on a transverse section (*z* = + 60) of the MNI standard brain. **(d)** Averaged brain activity (parameter estimate) across participants in the button press (cyan) and stick-rotation tasks (orange) in the YA and OA groups. Error bars indicate SEM. **(e)** Temporal profile of brain activity at a sphere with a 4-mm radius around the peak of M1 deactivation [(36, - 18, 46)] during the stick-rotation task in the YA group. Left, YA group; right, OA group. The red area indicates the stick-rotation task epoch. The x-axis represents the time course in which the start time of the task epoch is set to 0 s. The y-axis indicates the brain activity level (a.u.). Gray shaded regions in the graph indicate SEM. MNI, Montreal Neurological Institute; YA, younger adult; OA, older adult; a. u., arbitrary unit; SEM, standard errors of the mean across participants.

As our primary interest was in the bilateral sensorimotor cortices, we defined ROIs in the hand/finger sections of the bilateral PMd, M1, S1, and Area 2. In defining ROIs, we combined publicly available anatomical maps with a functional image from an independent experiment in which 29 healthy right-handed younger adults performed 60° flexion–extension of the left and right hand each at 1 Hz (Supplementary Information). We created anatomical maps for M1 (areas 4a, 4p), S1 (areas 3a, 3b, and 1), and Area 2 using cytoarchitectonic probability maps of the MNI standard brain in the SPM anatomy toolbox v3.0 (Amunts et al., 2020; Eickhoff et al., 2005). In the current cytoarchitectonic maps, the PMd was limited to the medial aspect (areas 6d1, 6d2, and 6d3). We used the precentral map in the Harvard–Oxford cortical map (Desikan atlas) for the anatomical definition of PMd (Desikan et al., 2006). We determined the hand/finger sections of M1, S1, and Area 2 in each hemisphere by depicting the overlap between the functional map and each cytoarchitectonic map. Similarly, we defined the hand/finger section of the PMd in each hemisphere by depicting the overlapping section between the functional map and Desikan’s precentral map, excluding M1 and S1 ROIs. The defined PMd ROI was located within the preliminary cytoarchitectonic map of area 6 (Ehrsson et al., 2003). Using this procedure, we consequently defined an ROI for the left or right PMd, M1, S1, and Area 2 (Figure 2a). The total number of voxels was 996, 469, 393, and 308 for the left PMd, M1, S1, and Area 2 ROIs, respectively, and 896, 312, 555, and 295 for the right PMd, M1, S1, and Area 2 ROIs, respectively. In functional connectivity analysis, we used these ROIs to detect significant brain activity and define seed regions.

#### 2.4.4 Contrast analysis (second-level)

For the second-level analysis, we used a full factorial design with a within-participant factor [task (2): button-press task, stick-rotation task] and a between-participant factor [group (2): YA, OA]. We first identified activation and deactivation during the button-press and stick-rotation tasks in each group (Figure 2b) before assessing differences between tasks (stick rotation > button press) in each group (Supplementary Figure 1) and the interaction between tasks and groups [(stick rotation > button press in OA) > (stick rotation > button press in YA); Figure 2c].

In these analyses, to identify significant activation and deactivation, we separately used a small volume correction (SVC) in contralateral (the left PMd, M1, S1, and Area 2) and ipsilateral ROIs (the right PMd, M1, S1, and Area 2). We adopted a family-wise error rate (FWE)-corrected extent threshold of *p* < 0.05 for a voxel-cluster image using an uncorrected voxel-wise threshold of *p* < 0.005, which we used consistently. We used cytoarchitectonic probability maps for anatomical identification of activation and deactivation peaks (Amunts et al., 2020; Eickhoff et al., 2005).

##### 2.4.4.1 Validation of contrast analysis results

To verify activation and deactivation in each task and group, we counted the number of activated and deactivated voxels in each ROI (Supplementary Figure 2).

Additionally, we extracted individual brain activity (parameter estimates) from the significant M1 cluster and showed the average for each task and group to visualize the interaction effect as we did for the significant interaction in the right M1 [(stick rotation > button press in OA) > (stick rotation > button press in YA)] (Figure 2d).

Furthermore, we carefully investigated the temporal profile of brain activity in the ipsilateral M1 during button-press (Supplementary Figure 3) and stick-rotation tasks (Figure 2e) in each group. We extracted time-course brain activity data from 13 volumes (for 30 s) immediately before, during, and immediately after each task epoch (for 15 s during the task and 7.5 s immediately before and after the task). We applied this to each participant for each of the first to fifth task epochs in each session. As there was no rest period following the final task epoch, we computed that segment as NaN. In each group, we extracted the time-course data using the CONN toolbox. The data were taken from a sphere with a 4 mm radius centered on the peak of the M1 deactivation identified in the YA group for each task (button-press: [40, –22, 66]; stick-rotation: [36, –18, 46]). For each participant, we averaged the time-course data related to the 10 task epochs (5 epochs × 2 sessions; before, during, and after) and calculated the grand average and standard error of the mean across all participants in each group. This descriptive analysis did not involve statistical analysis.

**FIGURE 3:**
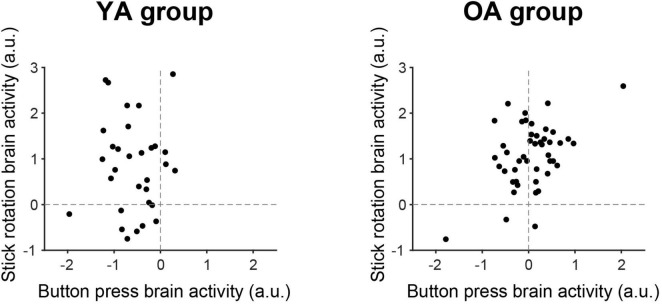
Relationship of brain activity between the button-press and stick-rotation tasks in the ipsilateral PMd, S1, and Area 2 clusters. In each participant, we extracted brain activity from the set of ipsilateral PMd and S1/Area 2 clusters (Figure 2b, top right) consistently observed during the stick-rotation task in both groups. We also extracted the activity from the same clusters during the button-press task and visualized the relationship of brain activity between the button-press and stick-rotation tasks across participants in each group. Left and right panels correspond to the YA and OA groups, respectively. x- and y-axes indicate brain activity during the button-press and stick-rotation tasks, respectively. YA, younger adults; OA, older adults; a. u., arbitrary unit.

Finally, we examined the relationship of brain activity between the button-press and stick-rotation tasks across participants (Figure 3). In each participant, we extracted brain activity from the set of ipsilateral PMd and S1/Area 2 clusters (Figure 2b, right top panel) consistently observed during the stick-rotation task in both groups. We also extracted the activity from the same clusters during the button-press task and visualized the relationship of brain activity between both tasks across participants in each group (Figure 3).

#### 2.4.5 Interparticipant correlation analysis

We examined whether the ipsilateral sensorimotor activity during the constant-paced stick-rotation task correlated with the individual performance capacity in the task (i.e., maximum stick rotation performance outside the MRI scanner). In this way, we tested the hypothesis that participants with lower performance capacity had higher ipsilateral sensorimotor activity to complement their clumsy performance. This approach is similar to that used in many previous studies investigating the relationship between brain activity patterns during certain tasks and personal traits evaluated using questionnaires. We performed a correlation analysis in each group using the performance capacity as a covariate. In this analysis, we excluded younger participants from the above behavioral analysis and used the SVC approach in the ipsilateral ROIs.

**FIGURE 4:**
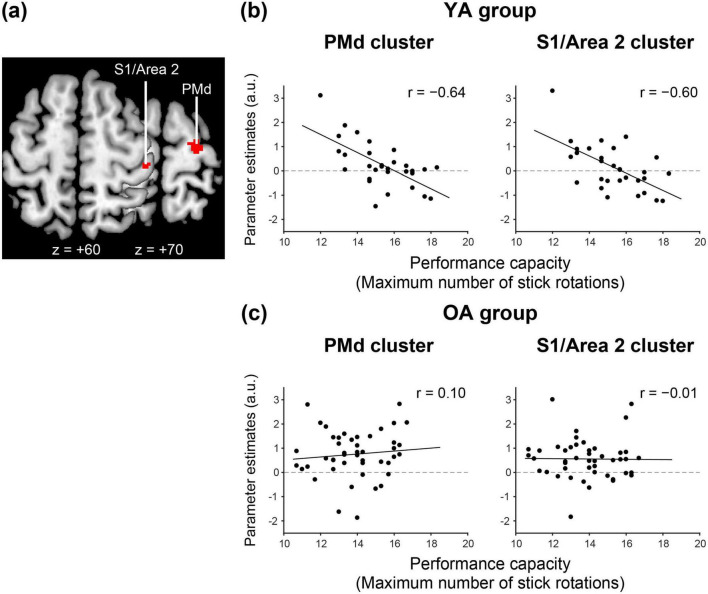
Brain regions in which activity is negatively correlated with performance capacity in stick rotation in the YA group **(a)**, and interparticipant correlation between brain activity and performance capacity in the YA **(b)** and OA **(c)** groups. **(a)** In the YA group, activities in the ipsilateral S1/Area 2 and PMd (red sections) during the 0.8 Hz stick-rotation task are negatively correlated with the performance capacity of stick rotation measured outside the scanner. These parameters are superimposed on the horizontal sections of z = + 60 and + 70 of the MNI standard brain. **(b,c)** Interparticipant correlation between performance capacity (x-axis) and brain activity (y-axis) in the YA **(b)** and OA **(c)** groups. Left, correlation between performance capacity and activity in the PMd cluster; right, correlation between performance capacity and activity in the S1/Area 2 cluster. Solid lines indicate linear regression lines fitted to the data. MNI, Montreal Neurological Institute; YA, younger adult; OA, older adult; a. u., arbitrary unit.

This analysis revealed significant PMd and S1/Area 2 clusters only in the YA group (Figure 4a). We extracted individual brain activity from each cluster and displayed the interparticipant correlation between brain activity and performance capacity (Figure 4b). In the OA group, we also displayed the interparticipant correlation using clusters identified in the YA group (Figure 4c). This aimed to allow for a visualization of the correlation results for both groups but was not statistically analyzed.

Moreover, we investigated brain regions whose activity during the 0.8 Hz stick-rotation task correlated with the individual performance capacity in the task in the contralateral ROIs (Supplementary Figure 4) and the whole brain (Supplementary Figure 5). We did not perform a correlation analysis between brain activity and button-press performance.

#### 2.4.6 Task-related functional connectivity analysis

**FIGURE 5:**
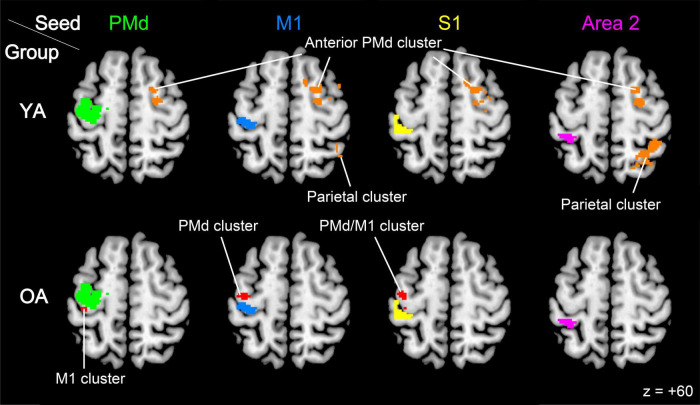
Brain regions in which activity increased functional connectivity with contralateral seed regions during the stick-rotation task compared with that during the button-press task in each group. The ipsilateral anterior PMd (orange sections) consistently enhanced functional coupling with all seed regions in the YA group (top row), whereas we observed brain regions (red sections) in which activity enhanced functional coupling with the PMd, M1, and S1 seeds within the contralateral ROIs in the OA group (bottom row). Green, blue, yellow, and magenta sections represent the contralateral seed regions PMd, M1, S1, and Area 2, respectively. The activities are superimposed on a transverse section (z = + 60) of the MNI standard brain. MNI, Montreal Neurological Institute; YA, younger adult; OA, older adult; PMd, dorsal premotor cortex; M1, primary motor cortex.

We conducted a generalized psychophysiological interaction (gPPI) analysis to identify brain regions in which activity showed enhanced functional coupling with contralateral seed regions (see below) during the stick-rotation task compared with that in the button-press task (McLaren et al., 2012). In this analysis, we preprocessed and analyzed the raw EPI images using the CONN toolbox version 20.b (Nieto-Castanon, 2020; Whitfield-Gabrieli and Nieto-Castanon, 2012). Physiological noise originating from the white matter and cerebrospinal fluid (CSF) was removed using the component-based noise correction method (CompCor) in the toolbox (Behzadi et al., 2007). Head motion-related artifacts, scrubbing, and condition effects were also removed. A temporal band-pass filter of 0.008–0.09 Hz was applied to examine task-related functional connectivity changes in a range of brain activity fluctuation slower than the cardiac and respiratory cycles (0.1–1.2 Hz) (Cordes et al., 2001). We prepared four seed regions in the contralateral PMd, M1, S1, and Area 2. First, we performed a conjunction analysis (uncorrected voxel-wise threshold of *p* < 0.005 and extent threshold of *p* < 0.05, corrected) (Price and Friston, 1997) to identify brain regions equally active between the YA and OA groups during the stick-rotation task. Each seed region was defined by identifying the overlapping region between this functional image and the left PMd, M1, S1, and Area 2 ROIs, respectively (Figure 5).

Each of the four seed regions was used for the gPPI analysis. For each participant, we deconvolved the time course of the average fMRI signal across voxels in each seed region using the canonical HRF (physiological variable). Subsequently, we performed a general linear model analysis using the design matrix and included the following regressors: the physiological variable, boxcar function for the task epoch (psychological variable), and multiplication of the physiological and psychological variables (PPI). We convolved these variables using a canonical HRF and included six realignment parameters in the design matrix as regressors of no interest.

For each task, we first generated an image of voxels showing the extent of activity changes with the PPI regressor of each seed region in each participant. Next, we generated a contrast image (stick rotation > button press) showing the stick-rotation task-related connectivity changes for each participant. We used this individual image in the second-level group analysis, including task order as a nuisance covariate, to exclude the potential influence of the factor on the results, as the order was counterbalanced across participants. In the second-level analysis, we separately searched for significant clusters in contralateral (the left PMd, M1, S1, and Area 2) and ipsilateral ROIs (the right PMd, M1, S1, and Area 2) in each group. In the YA group, no significant clusters were identified in the ROIs. However, because we found significant clusters in the entire brain in this group (in motor-related areas just outside the ipsilateral ROIs), we also reported these clusters (uncorrected voxel-wise threshold of *p* < 0.005 and extent threshold of *p* < 0.05, FWE-corrected; Figure 5). In addition, we conducted a correlation analysis to examine whether the change in stick-rotation task-related connectivity was correlated with individual performance capacity; however, no such regions were identified.

## 3 Results

### 3.1 Motor performance

In the button-press task, both groups successfully performed the 1-Hz button pressing inside the scanner. The CV of the button pressing interval—a measure of performance of the button-press task—was 0.072 ± 0.021 and 0.076 ± 0.018 in the YA and OA groups, respectively (Figure 1b, left panel), showing no significant between-group differences [*t*(54.23)=−0.90, *p* = 0.37, Bonferroni-adjusted α = 0.025, 95% Confidence intervals (CI) for the mean difference: (–0.01, 0.01), Hedges’ g* = –0.213, Welch’s *t*-test]. These results confirm that the performance of the button-press task was comparable between the two groups.

In the stick-rotation task, we visually confirmed that both groups could perform the 0.8-Hz stick rotation in the scanner. When we evaluated the performance capacity of stick rotation (i.e., maximum number of stick rotation in 10 s) outside the scanner, the stick rotation was performed 15.37 ± 1.64 and 13.86 ± 1.66 times over 10 s in the YA and OA groups, respectively (Figure 1b, right panel). Welch’s *t*-test revealed a significantly higher performance capacity in the YA group than in the OA group [*t*(62.57) = 3.89, *p* = 2.0 × 10^–4^, Bonferroni-adjusted α = 0.025, 95% CI for the mean difference: [0.62, 2.39], Hedges’ g* = 0.902].

### 3.2 Activation and deactivation in the YA and OA groups

In the YA group, all areas (PMd, M1, S1, and Area 2) in the ipsilateral ROIs were deactivated during the button-press task as expected (Figure 2b, top left; Supplementary Figure 3, left panel); however, the PMd, S1, and Area 2 in ipsilateral ROIs were activated during the stick-rotation task, whereas the M1 remained deactivated, similar to that in the button-press task (Figure 2b, top right). In the contralateral ROIs, the button-press task activated PMd/M1 and deactivated Area 2, whereas the stick-rotation task activated all areas.

In the OA group, unlike the YA group, significant deactivation was not observed in ipsilateral ROIs during the button-press task as expected (Figure 2b, bottom left; Supplementary Figure 3, right panel). Moreover, this difference was observed even when the OA group showed comparable button-press performance to the YA group (Figure 1b, left). The stick-rotation task activated all areas in the ipsilateral ROIs (Figure 2b, bottom right). This indicates that the deactivation of the ipsilateral M1 in the YA group (Figure 2b, top right) disappeared in the OA group (Figure 2b, bottom right), consistent with the temporal profile of ipsilateral M1 activity during the stick-rotation task (Figure 2e). Finally, both tasks activated all areas in the contralateral ROIs.

Overall, the stick-rotation task more strongly activated all areas in the ipsilateral ROIs than the button-press task in the OA group, and the former task only activated the ipsilateral PMd, S1, and Area 2 in the YA group (Supplementary Figure 1; Supplementary Table 1).

Table 1 summarizes the activation and deactivation peaks. We validated these results by counting the activated and deactivated voxels in each ROI (Supplementary Figure 2).

**TABLE 1:** Activation and deactivation during button-press and stick-rotation tasks within bilateral regions-of-interest (ROIs) in each group.

	Contralateral ROIs												Ipsilateral ROIs											
	Activation						Deactivation						Activation						Deactivation					
	Size	*t*-value	x	y	z	Anatomical identification	Size	*t*-value	x	y	z	Anatomical identification	Size	*t*-value	x	y	z	Anatomical identification	Size	*t*-value	x	y	z	Anatomical identification
**YA group**																								
*Button* *Press*	66	3.61	−36	−18	54	PrG	203	4.35	−20	−42	64	Area 2							1,751	7.07	40	−22	66	PrG
								4.09	−26	−38	58	Area 5L (SPL)								6.21	42	−34	58	Area 2
								3.87	−36	−32	46	PoG								5.88	34	−18	46	Area 4p
*Stick* *Rotation*	1,755	13.02	−44	−16	60	PrG							233	6.70	56	−18	46	Area 1	476	6.84	36	−18	46	Area 4p
		12.09	−40	−20	54	Area 4a								6.18	42	−36	56	PoG		6.50	28	−24	54	PrG
		9.21	−36	−14	66	Area 6d1								5.70	36	−34	46	Area 2		4.00	18	−24	76	Area 6d1
													194	5.37	42	−6	60	PrG						
														4.16	26	−8	68	Area 6d1						
**OA group**																								
*Button* *Press*	1,386	6.65	−40	−20	50	Area 4p																		
		6.43	−54	−20	42	Area 3b																		
		6.40	−32	−18	52	PrG																		
*Stick* *Rotation*	2,123	11.16	−44	−22	48	Area 3b							1,670	9.17	58	−18	42	Area 2						
		10.79	−46	−24	60	Area 1								7.91	32	−14	64	PrG						
		10.79	−32	−24	50	Area 4p								6.88	42	−26	48	Area 3b						

Height threshold, *p* < 0.005, uncorrected; extent threshold, *p* < 0.05, separately FWE-corrected within the contralateral and ipsilateral ROIs using SVC. Size refers to the number of significant voxels. For anatomical peak identification, we considered only cytoarchitectonic areas available in the anatomy toolbox with > 30% probability. We reported the cytoarchitectonic area with the highest probability for each peak. When cytoarchitectonic areas with > 30% probability were unavailable, we simply provided the anatomical peak location. In each cluster, we reported only peaks > 8 mm apart in order of larger *t*-values. To facilitate visualization, we avoided reporting a peak for each cluster identified in the cytoarchitectonic area or anatomical structure already reported for a peak with a higher *t*-value. FWE, family-wise error rate; PrG, precentral gyrus; PoG, postcentral gyrus; SPL, superior parietal lobule; SVC, small volume correction.

When testing the interaction [(stick rotation > button press in OA) > (stick rotation > button press in YA)], we found a significant M1 cluster (223 voxels; peak coordinates=28, −24, 54) in the ipsilateral ROIs, but none in the contralateral ROIs. This may be because, in the OA group, the ipsilateral M1 was activated during the stick-rotation task, whereas in the YA group, it remained deactivated (Figures 2c,d; Table 2).

**TABLE 2:** Areas showing significant interactions between task and group.

Size	*t*-value	x	y	z	Anatomical identification
223	4.72	28	−24	54	PrG
	3.72	34	−18	48	Area 4p
	3.56	26	−32	62	Area 3b

Height threshold, *p* < 0.005, uncorrected; extent threshold, *p* < 0.05, FWE-corrected within the contralateral and ipsilateral ROIs separately, using SVC. Size refers to the number of significant voxels. For anatomical peak identification, we considered only cytoarchitectonic areas available in the anatomy toolbox with > 30% probability. We reported the cytoarchitectonic area with the highest probability for each peak. When cytoarchitectonic areas with > 30% probability were unavailable, we simply provided the anatomical location of the peak. In each cluster, we reported only peaks > 8 mm apart in order of larger *t*-values. To facilitate visualization, we avoided reporting a peak for each cluster when it was identified in the cytoarchitectonic area or anatomical structure already reported for a peak with a higher *t*-value. PrG, precentral gyrus; SVC, small volume correction.

Finally, when we examined the relationship of brain activity in the ipsilateral PMd, S1, and Area 2 between the button-press and stick-rotation tasks across participants (Figure 3), most younger participants (27 of 31) showed deactivation during the button-press task and activation during the stick-rotation task (Figure 3, left panel). In contrast, almost half of the older participants (26 of 46) showed activation (instead of deactivation) during the button-press task, and most (43 of 46) showed activation during the stick-rotation task (Figure 3, right panel).

### 3.3 Brain regions in which activity correlated with performance capacity during stick rotation

We examined brain regions in the ipsilateral ROIs in which activity during the (constant-paced) stick-rotation task correlated with the performance capacity of stick rotation outside the scanner.

In the YA group, we found two clusters of voxels in which activity was negatively correlated with performance capacity. One was in the PMd, and the other was in S1/Area 2 (Figure 4a; Table 3). These regions partially overlapped with regions active during the stick-rotation task (Figure 2b, top right). Brain activity in both clusters increased in individuals with lower performance capacity measured outside the scanner (Figure 4b).

**TABLE 3:** Brain regions in which activity was correlated with performance capacity in the stick-rotation task.

Size	*t*-value	x	y	z	Anatomical identification
47	4.74	24	−16	70	Area 6d1
58	3.80	44	−22	50	Area 2
	3.35	40	−18	44	Area 3b
	3.22	44	−20	58	Area 1

Height threshold, *p* < 0.005, uncorrected; extent threshold, *p* < 0.05, FWE-corrected within ipsilateral ROIs using SVC. Size refers to the number of significant voxels. For anatomical peak identification, we considered only cytoarchitectonic areas available in the anatomy toolbox with > 30% probability. We reported the cytoarchitectonic area with the highest probability for each peak. When cytoarchitectonic areas with > 30% probability were unavailable, we simply provided the anatomical peak location. In each cluster, we reported only peaks > 8 mm apart in order of larger *t*-values. To facilitate visualization, we avoided reporting a peak for each cluster identified in the cytoarchitectonic area or anatomical structure already reported for a peak with a higher *t*-value. FWE, family-wise error rate; SVC, small volume correction.

In contrast, in the OA group, although all areas in the ipsilateral ROIs (including the M1) were activated during the stick-rotation task (Figure 2b bottom right), none showed such a significant correlation with performance capacity (Figure 4c). Furthermore, no region showed a significant correlation with performance capacity in the whole brain in this group.

In the contralateral ROIs, S1/Area 2 activity was negatively correlated with performance capacity in the YA group (Supplementary Figure 4), which was not observed in the OA group. No regions showed a positive correlation with performance for either the ROI or the group. Other regions (foot section of bilateral M1/SMA and the left area hOc4lp) in the whole brain (outside of the bilateral sensorimotor ROIs) where activity was negatively correlated with performance capacity in the YA group are shown in Supplementary Figure 5.

### 3.4 Enhanced functional connectivity during the stick-rotation task in the YA and OA groups

We examined brain regions within the contralateral or ipsilateral ROIs where activity increased functional coupling with each seed region (the contralateral PMd, M1, S1, or Area 2) during the stick-rotation task compared with that during the button-press task. In the YA group, no significant clusters were identified within either ROI. However, when we searched for clusters in the entire brain, we identified significant clusters just outside the ipsilateral ROIs in motor-related areas (Figure 5, top row). The anterior part of the ipsilateral PMd (partially overlapping with the ipsilateral PMd ROI) enhanced interhemispheric functional coupling consistently with all seed regions during the stick-rotation task compared with that during the button-press task (Figure 5, top row). Similarly, the ipsilateral intraparietal sulcus area, superior parietal lobule (SPL), and inferior parietal lobule just posterior to Area 2 enhanced interhemispheric functional coupling with the contralateral M1 and Area 2 (Figure 5, top row; Table 4).

**TABLE 4:** Functional connectivity results (stick rotation > button press).

Seed	Cluster	Size	*t*-value	x	y	z	Anatomical identification
**YA group**							
*PMd*	Anterior PMd cluster	227	5.01	20	−6	62	Area 6d3
			4.06	20	14	62	Area 6d2
*M1*	Parietal cluster	135	4.68	44	−58	52	Area PGa (IPL)
			3.28	48	−52	44	Angular Gyrus
			3.16	40	−48	60	Area 7PC (SPL)
	Anterior PMd cluster	178	4.61	20	2	62	Area 6d2
			3.89	34	−6	62	PrG
			3.80	20	−8	62	Area 6d1
*S1*	Anterior PMd cluster	238	5.43	24	−8	50	Area 6d3
			4.54	16	6	62	Area 6d2
			3.98	24	−8	66	Area 6d1
*Area2*	Anterior PMd cluster	292	7.35	24	−8	50	Area 6d3
			5.54	26	−10	62	Area 6d1
			4.96	24	−2	68	Area 6d2
	Parietal cluster	213	5.69	28	−52	62	Area 7PC (SPL)
			3.93	26	−64	56	Area 7A (SPL)
**OA group**							
*PMd*	M1 cluster	117	5.01	−40	−26	54	Area 4p
			3.44	−42	−16	56	PrG
*M1*	PMd cluster	37	5.10	−38	−16	60	PrG
*S1*	PMd/M1 cluster	89	5.21	−40	−16	62	PrG
			4.31	−36	−20	52	Area 4p

The brain regions identified in the YA group were in motor-related areas immediately outside the ipsilateral ROIs, whereas those in the OA group were within the contralateral ROIs. Height threshold, *p* < 0.005, uncorrected; extent threshold, *p* < 0.05, FWE-corrected across the entire brain in the YA group and within the contralateral and ipsilateral ROIs separately, using SVC in the OA group. Size refers to the number of significant voxels. For anatomical peak identification, we considered only cytoarchitectonic areas available in the anatomy toolbox with > 30% probability. We reported the cytoarchitectonic area with the highest probability for each peak. When cytoarchitectonic areas with > 30% probability were unavailable, we simply provided the anatomical peak location. In each cluster, we reported only peaks > 8 mm apart in order of larger *t*-values. To facilitate visualization, we avoided reporting a peak for each cluster identified in the cytoarchitectonic area or anatomical structure already reported for a peak with a higher *t*-value. We also reported only sensorimotor-related areas in the YA group. FWE, family-wise error rate; IPL, inferior parietal lobule; OA, older adult; PrG, precentral gyrus; SPL, superior parietal lobule; SVC, small volume correction; YA, young adult.

Unlike the YA group, in the OA group, we observed significant clusters only within the contralateral ROIs. The M1 exhibited enhanced functional coupling with the contralateral PMd during the stick-rotation task compared with that during the button-press task. Similarly, the contralateral PMd enhanced the functional coupling with the M1. Finally, the contralateral PMd and M1 exhibited increased functional coupling with the S1. We did not identify any significant clusters within ipsilateral ROIs (Figure 5, bottom row; Table 4) and in the aforementioned motor-related areas. Overall, functional connectivity (rather than interhemispheric connectivity) increased locally within the contralateral hemisphere in the OA group.

## 4 Discussion

### 4.1 Younger adults

During the button-press task, the ipsilateral sensorimotor cortices were widely deactivated (Figure 2b, top left). Ipsilateral sensorimotor deactivation during simple sensory and motor tasks is well documented and is considered to be caused by interhemispheric inhibition from the contralateral cortices (see Introduction). The robust ipsilateral sensorimotor deactivation observed during the button-press task in this study (Figures 2b top panel, 3; Supplementary Figure 3) aligns with this view.

During the stick-rotation task, the ipsilateral sensorimotor cortices were activated (Figure 2b top right) similar to those in previous studies investigating the neural correlates of dexterous and demanding motor tasks (Andrushko et al., 2021; Barany et al., 2020; Buetefisch et al., 2014; Hutchinson et al., 2002; Loibl et al., 2011; Nambu et al., 2015; Uehara et al., 2012, 2019; Verstynen et al., 2005). However, these activities were observed primarily in the PMd, S1, and Area 2, while the ipsilateral M1 remained deactivated, as it was during the button-press task. Hence, our study elucidated clear regional differences in ipsilateral sensorimotor activation during the stick-rotation task. In the present study, we used a relatively small (4-mm FWHM) Gaussian filter to spatially smoothen functional images. However, this is not the source of regional differences because we observed the same pattern of ipsilateral activation and deactivation when using the larger filter (8 mm), which was often used in previous studies (Supplementary Figure 6). In addition, the brain activity measured when another nine healthy right-handed younger adults performed the same stick-rotation task indicated that all consistently showed ipsilateral M1 deactivation, seven participants showed ipsilateral PMd, and five showed ipsilateral S1/Area 2 activations (Supplementary Figure 7). At the group level, we observed ipsilateral PMd activation and M1 deactivation, replicating the current findings (Supplementary Figure 7). Thus, the pattern of ipsilateral activation and deactivation (i.e., regional difference) in the stick-rotation task appears highly reproducible.

Finally, most younger participants exhibited deactivation in the ipsilateral PMd, S1, and Area 2 during the button-press task and activation during the stick-rotation task, particularly those with lower performance capacity (Figure 3, left panel; Figure 4b). Younger adults with lower performance capacity appeared to perform the stick-rotation task at 0.8 Hz by increasing the ipsilateral activity, probably by disinhibiting the interhemispheric inhibition operating during the button-press task, whereas those with higher performance capacity could perform the stick-rotation task without increasing the ipsilateral activity (Figure 4b). Hence, the ipsilateral sensorimotor activity during the dexterous hand motor task could indicate a brain complement mechanism for its poor performance, whereas the ipsilateral sensorimotor deactivation could indicate better performance.

#### 4.1.1 M1

We adopted the current stick-rotation task as a dexterous task because it requires fine control and coordination of individual finger movements to manipulate the stick precisely. This type of task reproducibly deactivated the ipsilateral M1 (Figure 2b top right; Supplementary Figure 7). These findings indicate that tasks requiring finger dexterity do not necessarily require ipsilateral M1 activity. Although the current task deactivated the ipsilateral M1, ipsilateral M1 activation has been reported during other types of demanding hand motor tasks—for instance, high-force unimanual handgrip (Andrushko et al., 2021; Barany et al., 2020; Buetefisch et al., 2014; see Introduction). Hence, the recruitment of the ipsilateral M1 may be task-dependent. In typically developed younger adult brains, interhemispheric facilitatory and inhibitory circuits coexist between the left and right M1s (Ni et al., 2020). Hence, younger adult brains can adaptively control various movements by flexibly and plastically changing the interhemispheric facilitation and inhibition balance between the two M1s.

Ipsilateral M1 inhibition is an important factor for higher finger dexterity. Previous studies have shown lower finger dexterity in children with immature ipsilateral M1 inhibition and older adults with deteriorated ipsilateral M1 inhibition when compared with that in younger adults with mature ipsilateral M1 inhibition (Naito et al., 2020, 2021). In addition, age-related decline in right-hand dexterity could improve through 2-month bimanual movement training, which could reactivate interhemispheric inhibition that has deteriorated with age (Naito et al., 2021). Consistent with these views, we observed ipsilateral M1 deactivation during the stick-rotation task in the YA group (Figure 2b, top right), who had a relatively higher performance than the OA group (Figure 1b, right panel). However, we did not find a correlation between the degree of M1 deactivation and performance capacity across participants, similar to that in our previous study (Naito et al., 2020). With mature ipsilateral M1 inhibition, the degree of M1 deactivation does not apparently correlate with performance capacity in a dexterous finger motor task.

The temporal profile of the ipsilateral M1 deactivation (negative BOLD) suggests that suppression of brain activity primarily occurs in the first half of the stick-rotation task, starting around task initiation (Figure 2e, left panel). While the neurophysiological basis for a negative BOLD is not fully understood, a recent human study has suggested its association with the suppressed increase in neural activity mediated by GABA-ergic inhibition (Fracasso et al., 2022). Hence, a negative BOLD signal might reflect a neural state that decreases the likelihood of increased neuronal firing in the deactivated region.

The current stick-rotation task deactivated ipsilateral M1 from the beginning of the task. A TMS study in which young adults performed fine control and coordination of individual finger movements to manipulate chopsticks has shown that the excitability of the M1 ipsilateral to the hand increases when single-pulse TMS is given 3 ms after conditioning TMS to the ipsilateral M1 during the task (Morishita et al., 2011). This phenomenon could represent a facilitative effect, possibly due to the enhancement of the facilitatory input from the contralateral to the ipsilateral M1 (Ni et al., 2020), which is similar to the motor overflow effect (Hoy et al., 2004). The present negative BOLD signal might reflect neural states where this facilitatory effect in the ipsilateral M1 is suppressed primarily in the first half of the stick-rotation task from task initiation (Figure 2e, left panel). However, these speculations require further studies to elucidate the neurophysiological basis of negative BOLD signal and bridge the gap between fMRI and electrophysiology, as a negative BOLD response is typically delayed from its underlying neural activity (Shmuel et al., 2006).

#### 4.1.2 PMd

In precentral motor regions, while the ipsilateral M1 was deactivated, the ipsilateral PMd was activated during the stick-rotation task (Figure 2b, top right). Moreover, as we hypothesized, participants with lower performance capacity of stick rotation recruited more ipsilateral PMd activity to perform the 0.8 Hz stick rotation (Figures 4a, b left panel). This rebuffs the view that the activity increase deteriorates performance because they could perform the 0.8-Hz stick rotation as well as those with higher performance capacity. A plausible interpretation is that the 0.8-Hz stick rotation could be more demanding for these participants than for those with higher performance capacity, and they recruited the PMd activity to complement their clumsy performance.

As described in the Introduction, the complementary role of the ipsilateral PMd in the control of hand movement is well documented in the brains of patients with stroke (Bestmann et al., 2010; Johansen-Berg et al., 2002; Lotze et al., 2006) and after spinal cord injury in non-human primates (Chao et al., 2019). In addition, corticospinal projection from the ipsilateral PMd is known in primates (Kuypers and Brinkman, 1970; Morecraft et al., 2019). Although the causal relationship between ipsilateral PMd activity and stick rotation performance requires further studies, the above lines of evidence imply that ipsilateral PMd in healthy younger adults could complement dexterous motor control. If correct, ipsilateral PMd recruitment could be a common strategy for the brain to compensate and complement hand motor function not only after spinal cord injury and brain stroke but also when healthy younger brains perform dexterous and demanding hand movements.

In the present study, the importance of the ipsilateral PMd during the stick-rotation task was corroborated by the finding that the ipsilateral PMd (anterior part) enhanced functional coupling consistently with all contralateral seed regions during the stick-rotation task (Figure 5, top row). This suggests that—among the ipsilateral sensorimotor cortices—the PMd is a particularly important region when the contralateral sensorimotor cortices try to communicate with the ipsilateral hemisphere during dexterous tasks. The cluster was located primarily anterior to the region active during the stick-rotation task (Figure 2b, top right) and to that where activity correlated with performance (Figure 4a). The anterior PMd region corresponds relatively well with that involved in higher-order motor planning/preparation, working in coordination with the SPL (Furuta et al., 2024; Gerardin et al., 2000; Hanakawa et al., 2008; Solodkin et al., 2004; Stephan et al., 1995). Thus, this anterior region may play a slightly different role than the regions where activity increased during the stick-rotation task (Figures 2b, 4a). For example, access from the contralateral sensorimotor cortices to the ipsilateral PMd may be associated with careful preparation for the stick-rotation task. Disregarding speculation, these results support our hypothesis that the PMd plays a prominent role among the ipsilateral sensorimotor cortices of younger adults performing dexterous finger movements.

#### 4.1.3 S1 and Area 2

During the stick-rotation task, ipsilateral S1 and Area 2 were activated as well (Figure 2b, top right). The current stick-rotation task involved three fingers. Thus, the brain likely received more somatosensory input from the hand/finger muscles and skin than during the simple button-press task with the index finger. In primates, Area 2 neurons are characterized by their involvement in the processing of somatosensory information from both hands (Iwamura et al., 1994), and the human Area 2 in each hemisphere responds to kinesthetic stimulation in both hands (Naito et al., 2005). Hence, ipsilateral S1 and Area 2 activation together with contralateral activation (Figure 2b, top right) during the stick-rotation task may be involved in similar complex somatosensory information processing. In agreement with this view, the ipsilateral (Figure 4b, right panel) and contralateral (Supplementary Figure 4) S1/Area 2 activity was negatively correlated with the performance capacity in stick rotation, i.e., activity increased in clumsy participants with lower stick rotation performance capacity. If the 0.8-Hz stick-rotation task was particularly demanding for these participants, the miscellaneous somatosensory input derived from redundant movements due to clumsy control of stick rotation might have increased these activities. However, given that jamming TMS to the SPL (Area 2) has been shown to disrupt finger movements (Lotze et al., 2006), the observed somatosensory activity may also reflect its involvement in motor-control processes. This could include sensory guidance (Rothwell et al., 1982) and/or sensory prediction (Christensen et al., 2007).

#### 4.1.4 Causality analysis using the linear non-Gaussian acyclic model (LiNGAM)

In the YA group, the stick-rotation task activated the contralateral sensorimotor cortices and the ipsilateral PMd, S1, and Area 2, whereas the ipsilateral M1 remained deactivated (Figure 2b, top right). In addition, the ipsilateral PMd (particularly anterior part) enhanced functional coupling consistently with all contralateral seed regions during the stick-rotation task (Figure 5, top row). However, these analyses do not provide information about the causal relationship between sensorimotor activities. Therefore, we performed causality analysis using LiNGAM to explore the causal relationship between brain activities across the eight bilateral ROIs (left or right PMd, M1, S1, or Area 2) during the stick-rotation task in the YA group (see Supplementary Information; Supplementary Figure 8). LiNGAM allows for the exploration of causal relationships (both positive and negative) between brain activities across multiple brain regions without prior knowledge or specific hypotheses regarding the network structure (Ogawa et al., 2022). Its drawback is that not all causal relationships can be clearly interpreted based on current neuroscientific knowledge.

The results are presented in Supplementary Figure 9. The connectivity analysis showed that the ipsilateral PMd enhanced functional coupling with all contralateral sensorimotor cortices (Figure 5, top row); however, in LiNGAM, the ipsilateral PMd received positive influences from the contralateral PMd and M1 (Supplementary Figure 9b). Consistent with the connectivity findings (Figure 5, top row), LiNGAM showed a positive influence of the contralateral PMd on the ipsilateral PMd. This finding is consistent with the observation in non-human primates that interhemispheric PMd–PMd interaction plays a crucial role when the brain compensates for a damaged contralateral motor pathway during the recovery phase of grasping after unilateral spinal cord injury (Chao et al., 2019). LiNGAM further showed that the ipsilateral M1, which was suppressed during the stick-rotation task (Figure 2b, top right), was ranked lower in causal order among all eight ROIs (Supplementary Figure 9b). The ipsilateral M1 was positively influenced by the ipsilateral PMd and negatively (inhibitory) influenced by the contralateral side (Area 2). Several studies have reported ipsilateral PMd and M1 activation during dexterous finger movements (Loibl et al., 2011; Uehara et al., 2012; Verstynen et al., 2005). LiNGAM indicated a hierarchical order in their recruitment: the PMd would be recruited almost immediately, but whether the M1 is recruited or not might depend on the interaction between the positive influence from the ipsilateral PMd and the negative influence from the contralateral sensorimotor cortices.

We also performed LiNGAM for data in the OA group; however, we could not find any significant causal relationships among current ROIs (see more discussion in Supplementary Information).

### 4.2 Older adults

Broader ipsilateral sensorimotor deactivation as observed in the YA group was not observed in the OA group during the button-press task (Figure 2b, bottom left). At the individual level, over 50% of older participants (26 of 46) showed activation instead of deactivation (Figure 3, right panel). The observed reduction in and/or loss of ipsilateral sensorimotor deactivation is consistent with those in previous reports (see Introduction). Similar pattern has also been reported during non-motor kinesthetic stimulation of the unilateral hand (Naito et al., 2021), suggesting that this phenomenon can occur independently of motor control and may simply reflect age-related reduction in interhemispheric inhibition. Although the neural mechanisms underlying this phenomenon remain unknown, if ipsilateral sensorimotor deactivation is associated with local neural inhibition mediated by an inhibitory neurotransmitter (GABA), an age-related decrease in GABA concentration (Gao et al., 2013) could explain the age-related reduction or loss of ipsilateral sensorimotor deactivation.

Age-related decline of inhibitory function in older adults is not limited to motor-cortical interhemispheric inhibition. A previous study (Morita et al., 2021) has shown that other types of inhibition—such as cross-somatotopic inhibition (Zeharia et al., 2012; Nakata et al., 2019), cross-modal inhibition (Lewis et al., 2000; Jorge et al., 2018), and inhibition within the default mode network (Marchand et al., 2007; Nakata et al., 2019; Kudo et al., 2004)—are also reduced or lost in many older adults aged over 65. Hence, inhibitory functions occurring between brain regions seem to be generally declined even in healthy older adults, making it difficult for their brains to process information properly using inhibition.

Unlike the effect in the YA group, the stick-rotation task in the OA group activated all areas in ipsilateral ROIs, including the ipsilateral M1 (Figures 2b, bottom right, e). Nevertheless, none of these areas showed a correlation between brain activity and performance capacity (Figure 4c). The increased activity in the ipsilateral sensorimotor cortices in the OA group was thus unrelated to their performance capacity in stick rotation or to complementation for clumsy finger movements (Figure 4c). Hence, the increase in ipsilateral M1 activity during the stick-rotation task in the OA group (Figure 2c) may be an epiphenomenon resulting from reduced or lost inhibition from the left to the right M1. The reduction or loss of this interhemispheric inhibition in these older participants appears supported by our observation of involuntary movements of the left finger in several older adults while performing the stick-rotation task in the scanner, sometimes called mirror movement (Carson, 2005) or mirror overflow (Luo et al., 2022) due to weakened interhemispheric inhibition (Hoy et al., 2004). The brains of older adults, whose interhemispheric inhibition has already been reduced during the button-press task, are unable to disinhibit this inhibition in response to the stick-rotation task demand. As a result, they cannot effectively engage the ipsilateral sensorimotor cortices. Our previous study has shown that older adults with reduction in interhemispheric inhibition tend to exhibit decreased hand dexterity (Naito et al., 2021). Hence, reduction in interhemispheric inhibition can be a sign of poorer hand dexterity.

We may further discuss why no sensorimotor cortices showed correlation with performance capacity in older adults. One possibility is that, as the LiNGAM result suggested (see more discussion in Supplementary Information), excessive bilateral sensorimotor activities during the stick-rotation task in the OA group (Figure 2b, bottom right) might have caused noise and disrupted information transmission in the brain network, so that correlation was difficult to see. Another possible explanation would be less adaptability to environmental changes in older adults compared to younger adults. As described in Methods 2.2 (3), in the present study, body posture and visual conditions differed between the in-scanner and out-of-scanner tasks. Older adults could be less adaptable to such environmental changes. While younger adults tend to exhibit stable hand dexterity and consistent brain activity across different environments and task conditions, older adults may show greater variability in both performance and brain activation, making it difficult to maintain consistency under changing conditions.

The transcallosal fibers between the left and right M1s are quantitatively and qualitatively degraded in older adults (Fling and Seidler, 2012; Lebel et al., 2012; Ota et al., 2006; Strauss et al., 2019; Sullivan et al., 2006). Thus, age-related ipsilateral activity may be related to reduced transcallosal fibers. In an aging brain with degraded interhemispheric fibers, recruiting interhemispheric regions for dexterous motor tasks may not be an optimal strategy. Instead, aging brains tend to increase short-range functional connectivity within the contralateral sensorimotor cortices, which is a developmentally regressive strategy, as functional brain networks typically develop from a local (short-range) to more distributed (long-range) organization (Amemiya et al., 2019; Dosenbach et al., 2010; Fair et al., 2009).

Brains of patients with stroke often recruit ipsilateral (i.e., contralesional) sensorimotor cortices to compensate for damaged hand motor function (Grefkes and Fink, 2020). In general, older adults exhibit slower recovery of motor function compared to younger adults (Yoo et al., 2020). This could be partially explained by the current result that aging brains have a limited capacity of complementing hand motor function with motor-cortical interhemispheric strategy. Aging brains seem to use a strategy of recruiting intrahemispheric regions in the sensorimotor cortices. Hence, once a stroke occurs in the contralateral sensorimotor cortices, the local complementary strategy within the affected sensorimotor cortices may not adequately compensate for functionality.

#### 4.2.1 Hemispheric asymmetry reduction in older adults (HAROLD)

The ipsilateral M1 activation observed during the stick-rotation task in the OA group (Figure 2b, bottom right) can be broadly interpreted as a manifestation of the HAROLD phenomenon, reflecting reduced hemispheric asymmetry in the aging brain. However, unlike prefrontal HAROLD, ipsilateral M1 activation in older adults does not appear to improve motor task performance, although ipsilateral M1 activation after stroke contributes to the restoration of motor function (Grefkes and Fink, 2020).

Previous studies have indicated that age-related ipsilateral sensorimotor activation—likely resulting from reduced interhemispheric inhibition—does not enhance motor task performance but reflects diminished motor function. For example, it is shown in older adults that (1) ipsilateral sensorimotor activation remains constant even with increasing movement frequency (Riecker et al., 2006), (2) the activation is associated with longer reaction times (Langan et al., 2010), and (3) a reduction in interhemispheric inhibition is associated with decreased hand dexterity (Naito et al., 2021). Similarly, the present study showed no correlation between ipsilateral M1 activity and performance capacity. These lines of evidence suggest that ipsilateral sensorimotor activation does not necessarily complement motor tasks in healthy older adults. Thus, the HAROLD concept may not apply well to the motor domain.

### 4.3 Limitations

This study provided novel insights into how younger and older brains utilize the ipsilateral sensorimotor cortices when performing a dexterous finger motor task. However, this study has several limitations. First, we only showed an indirect correlation between brain activity during the 0.8-Hz stick-rotation task and performance capacity in stick rotation evaluated outside the scanner. This approach allows the assessment of differences in brain activity based on individual performance levels during the same motor task. However, it only provides an indirect correlation between brain activity and performance. A detailed behavioral analysis (e.g., kinematics of finger movements) during the stick-rotation task while scanning might have revealed a more direct relationship between brain activity and performance. Similarly, simultaneous electromyogram recording while scanning may provide more precise information about the relationship between brain and muscle activities during the task. Finally, the present study only showed a “correlation” between brain activity and performance, but not “causality.” In the future, we aim to elucidate the causal relationship between ipsilateral sensorimotor activity, particularly PMd, and the simultaneously measured detailed performance using various neuromodulation techniques.

## 5 Conclusion

The present study examined how healthy right-handed younger adult brains utilize the ipsilateral sensorimotor cortices (PMd, M1, S1, and Area 2), which are typically suppressed during simple motor task to perform a dexterous finger motor task, and its age-related changes. Using a simple button-press task and a dexterous stick-rotation task, we demonstrated that PMd is a key structure among the ipsilateral sensorimotor cortices when younger adult brains perform the stick-rotation task to complement their clumsiness, probably by disinhibiting the interhemispheric inhibition that was operating during the button-press task and by enhancing the functional coupling with the contralateral sensorimotor cortices. We also showed that the ipsilateral sensorimotor activity during the stick-rotation task in aging brains does not effectively play a complementary role and has a different physiological meaning from that in younger adult brains, probably because of their degraded interhemispheric inhibition. The findings of the present study advance our understanding of the use of the ipsilateral sensorimotor cortices for the dexterous control of finger movements in younger adult brains and their age-related changes.

## Data Availability

The raw data supporting the conclusions of this article will be made available by the author, without undue reservation.
