# Supplementary material for: Younger adult brain utilizes interhemispheric strategy via ipsilateral dorsal premotor cortex for fine control of dexterous finger movements, unlike the aging brain
Source: Front Aging Neurosci. 2025 Jul 21;17:1501011. doi: 10.3389/fnagi.2025.1501011 (PMC12318952; doi:10.3389/fnagi.2025.1501011)
Supplement: Supplementary file 1 [file Data_Sheet_1.docx]

**Supplementary Information for**

**Younger adult brain utilizes interhemispheric strategy via ipsilateral dorsal premotor cortex for fine control of dexterous finger movements, unlike the aging brain**

Gen Miura, Tomoyo Morita, Jihoon Park, Eiichi Naito

**1 Generation of functional images of hand/finger sections in the bilateral sensorimotor cortices to define the regions-of-interest (ROIs)**

We collected functional images while an independent group of 29 healthy right-handed younger adults (19 men, mean age 25.4 ± 8.2) continuously exerted 60º cyclic extension–flexion movements of both hands in synchronization with 1-Hz cyclic tones. We reused a device developed and used in our previous study (Morita et al., 2023) to control the range of wrist motion. We mounted a movable hand-rest on the device, on which the hand was fixed, indicating the wrist angle. We fixed two stoppers onto the device to control the wrist motion range, preventing the wrist from extending beyond the straight (0º) position and flexing beyond 60º, across task epochs and participants. The participants had to touch one of the stoppers (0º or 60º) alternatively with the hand-rest in synchronization with the 1-Hz audio tones while making controlled and continuous wrist extension–flexion movements. Left- and right-hand tasks were performed in separate experimental sessions.

We acquired functional images using T2*-weighted gradient echo-planar imaging (EPI) sequences on a 3.0-Tesla MRI scanner (Trio Tim; Siemens, Germany) equipped with a 32-channel array coil. Each volume comprised 44 slices (slice thickness, 3.0 mm; interslice inter-slice thickness, 0.5 mm) acquired in ascending order, covering the entire brain. We acquired successive images from the same slice at 2,500 ms time intervals. We used an echo time (TE) of 30 ms and a flip angle (FA) of 80º. The field of view (FOV) was 192 × 192 mm^2^ and the matrix size was 64 × 64 pixels. Voxel dimensions were 3 × 3 × 3.5 mm^3^ in the x-, y-, and z-axes, respectively. For each experimental run, we collected 65 volumes. Using the same scanner, we acquired a T1-weighted magnetization-prepared rapid gradient echo (MP-RAGE) image as an anatomical reference. The imaging parameters were as follows: repetition time (TR) = 1900 ms, TE = 2.48 ms, FA = 9º, FOV = 256 × 256 mm^2^, matrix size = 256 × 256 pixels, slice thickness = 1.0 mm, voxel size = 1 × 1 × 1 mm^3^, and 208 contiguous transverse slices.

We asked the participants to close their eyes, relax their entire body, produce no unnecessary movements, and focus on the assigned task while performing a task. Each participant completed a 160-s experimental run for each task. The run comprised five task epochs, each lasting 15 s. In each epoch, the participants continuously exerted cyclic movements for each task synchronized with the cyclic audio tones. Between each task epoch, we inserted a 15-s baseline (rest) period. We also included a 25-s baseline period in each run before the beginning of the first epoch. During the experimental run, we provided the participants with auditory instructions indicating the start of a task epoch (“three, two, one, start”), as well as a “stop” instruction generated by a computer to signal the end of each epoch. During rest periods, the participants heard the same cyclic audio tones; however, they did not generate any movement. We provided all auditory stimuli through MR-compatible headphones. An experimenter beside the scanner bed visually observed the proper performance of each task throughout the run.

The same image preprocessing was performed (see main text), and the normalized images were filtered using a Gaussian kernel with a full-width at half-maximum of 4 mm along the x-, y-, and z-axes. After single-subject analysis (see main text), we performed a second-level group analysis. We identified activation during the task (task > rest) using the same threshold of p <0.05, family-wise error rate (FWE) corrected for a voxel-cluster image with an uncorrected voxel-wise threshold of p <0.005. We performed this procedure separately for the left- and right-hand tasks. Our analysis revealed significant activation in the hand/finger section of the left sensorimotor cortices during the right-hand task and in the homologous section of the right sensorimotor cortices during the left-hand task. We used these cluster images to functionally define the hand/finger sections of the bilateral sensorimotor cortices.

**2 Percentage of activated and deactivated voxels in each ROI**

In the second-level group analysis, we generated a voxel-cluster image with an uncorrected voxel-wise threshold of p <0.005 with no extent threshold. We generated activation (task > rest) and deactivation (rest > task) maps for each task in each group. To compute the percentage, we counted the number of activated and deactivated voxels and divided the voxel number by the total number of voxels in each ROI (see main text). Supplementary Figure 2 shows the results, which matched well with those obtained from the contrast analysis (Figure 2b).

**3** **Temporal profile of brain activity during the button press task in the YA and OA groups** Apart from the temporal profile of brain activity during the stick rotation task in Figure 2e, we analyzed the temporal profile of brain activity during the button press task in the YA and OA groups. We extracted the time-course data from a sphere with a 4 mm radius around the peak of ipsilateral M1 deactivation ([40, −22, 66]) during the button press task in the YA group. Supplementary Figure 3 shows that the brain activity suppression started before task initiation and higher suppression in the YA group than in the OA group.

**4 Other brain regions whose activity was negatively correlated with performance capacity in the whole brain in the YA group**

We searched for brain regions where activity during the 0.8-Hz stick rotation task correlated with the individual performance capacity of stick rotation in the whole brain. Then, we performed the same correlation analysis but searched for such brain regions in the whole brain (We report brain regions outside the bilateral sensorimotor ROIs; Figure 2a). We adopted a FWE-corrected extent threshold of p < 0.05 for a voxel-cluster image using an uncorrected voxel-wise threshold of p < 0.005 in the whole brain (as in the main text). In this analysis, we excluded one younger participant excluded from the behavioral analysis (see main text). In the YA group, significant clusters were identified in the foot section of the bilateral M1/SMA (peak coordinate: [0, −20, 74]) and in the left hOc4lp (peak coordinate: [−30, −94, 0]). In the OA group, no significant clusters were found.

In the YA group, we extracted the brain activity in each cluster for each individual and displayed the interparticipant correlation between the brain activity and the performance capacity (Supplementary Figure 5a, b, left panels, respectively). For the OA group, we also displayed the interparticipant correlation using the clusters identified in the YA group (Supplementary Figure 5a and 5b, right panels, respectively). These were performed to visualize the results of the correlation for both groups, but no statistical analysis was performed.

Furthermore, we investigated the relationship of the brain activity in each identified cluster (the foot section of bilateral M1/SMA, the left hOc4lp) between the button press task and the stick rotation task across participants. We extracted the activity from each cluster in each task and participant and visualized the relationship of brain activity between the two tasks across participants in each group (Supplementary Figure 5c, d).

In the YA group, activity in the foot section of the bilateral M1/SMA (peak coordinate: [0, -20, 74] during the 0.8 Hz stick rotation task correlated with the performance capacity (Supplementary Figure 5a left). In fact, this region was deactivated during the button press task in most participants (29 of 31; Supplementary Figure 5c left). Such deactivation is called cross-somatotopical inhibition, wherein a movement of an effector (ex. hand or foot) suppresses the activity of somatotopical sections of other effectors (ex. foot or hand) in the bilateral sensorimotor cortices (Zeharia et al., 2012; Morita et al., 2021; Naito et al., 2021) to perform the former by preventing interference from the latter. As we observed in the ipsilateral PMd and S1/Area 2, participants with lower performance capacity increased the activity of the foot section. Since the foot section is not directly involved in the motor control of the hand, this may indicate that participants with less dexterity perform the task by tensing the leg/foot muscles (This is unnecessary to perform the task, but they recruited this activity to perform (complement) for their clumsy performance). On the other hand, participants with higher performance capacity did not need to release the cross-somatotopical inhibition. Taken together, the activation in the ipsilateral sensorimotor cortices and in the foot section of the M1 during a dexterous hand motor task can indicate brain mechanisms that complement and/or compensate for its poor performance, whereas their deactivation can indicate better performance.

In the OA group, the activity of this region increased during the stick rotation task in most participants but was not correlated with the performance capacity (Supplementary Figure 5a right). In addition, over half of the participants showed activation (instead of deactivation) during the button press task (Supplementary Figure 5c right), suggesting that cross-somatotopical inhibition from the hand to the foot section was degraded in these older participants (Morita et al., 2021).

Similarly, in the YA group, the activity in the left hOc4lp (peak coordinate: [-30, -94, 0]) during the 0.8 Hz stick rotation task correlated with the performance capacity (Supplementary Figure 5b left). Importantly, such a correlation was observed while this region was deactivated in almost all participants (30 of 31). The most striking difference from the ipsilateral PMd, S1/Area 2 (Figure 4) and the foot section (Supplementary Figure 5c left) was that this visual region was deactivated in almost all participants (30 of 31) during the stick rotation task, while nearly half of the participants showed activation during the burton press task. Thus, deactivation in this region became more robust during the dexterous motor task than during the simple motor task, a completely opposite phenomenon from that observed in sensorimotor regions. Visual deactivation during a motor task –cross-modal inhibition–, whereby the brain tries to suppress activity in regions irrelevant to perform a task to prevent interference from the irrelevant regions (Morita et al., 2019). A previous study has shown that when the visual cortices are more deactivated, healthy young adults can perform an audio-motor task better (Morita et al., 2019). Accordingly, participants with higher performance capacity showed greater visual deactivation during the 0.8-Hz stick rotation task. Viewed collectively, cross-modal inhibition in visual areas during a motor task indicates better performance.

**5 Reproducibility of the contrast result during the stick rotation task in the YA group using a spatial Gaussian filter of 8 mm FWHM**

To determine whether the contrast result observed during the stick rotation task in the YA group (ipsilateral PMd and S1/Area2 activations and ipsilateral M1 deactivation; Figure 2b) was affected by the size (4 mm FWHM) of the spatial Gaussian filter, we repeated the analysis using a spatial Gaussian filter of 8 mm FWHM. The result was perfectly replicated (Supplementary Figure 6). Hence, we established that the result (= clear regional difference in the ipsilateral sensorimotor activation and deactivation during the stick rotation task) was not affected by the spatial filter size.

**6 Reproducibility of the stick rotation task results**

To ensure reproducibility in the stick rotation task in the YA group, we measured brain activity in nine other healthy right-handed younger participants (9 men, mean age 24.1 ± 1.2) while they performed the same stick rotation task (0.8 Hz stick rotation).

We acquired functional images using T2*-weighted gradient EPI sequences on a 3.0-Tesla MRI scanner (Vida; Siemens, Germany) equipped with a 64-channel array head-neck coil. We used a multiband imaging technique (multiband factor, 3; Moeller et al., 2010). Each volume comprised 51 slices (slice thickness, 3.0 mm with no inter-slice thickness) acquired in an interleaved manner, covering the entire brain, at 1,000 ms intervals. We used a TE of 30 ms and a FA of 60º, with a FOV of 210 × 210 mm^2^ and a matrix size of 70 × 70 pixels. Voxel dimensions were 3 × 3 × 3 mm^3^ in the x-, y-, and z-axes, respectively.

As an anatomical reference, a T1-weighted MP-RAGE image was acquired using the same scanner. The imaging parameters were as follows: TR = 1900 ms, TE = 2.48 ms, FA = 9º, FOV = 256 × 256 mm^2^, matrix size = 256 × 256 pixels, slice thickness = 1.0 mm, voxel size = 1 × 1 × 1 mm^3^, and 208 contiguous sagittal slices.

Participants performed two 190-s sessions each. Each session comprised six task epochs, each preceded by a rest epoch (baseline state) of 15 s. In addition, we provided an extra 10 s before the first rest epoch for magnetization stabilization.

The same image preprocessing as described in the main text was performed and the normalized images were filtered using a Gaussian kernel with a full-width at half-maximum of 4 mm along the x-, y-, and z-axes. We performed a single-subject analysis wherein we used a small volume correction approach to separately identify significant activation and deactivation in the contralateral ROIs comprising the left PMd, M1, S1, and Area 2, and in the ipsilateral ROIs comprising the right PMd, M1, S1, and Area 2. We reported activation (task > rest) and deactivation (rest > task) using an uncorrected voxel-wise threshold of p < 0.005 and an extent threshold of p < 0.05, FDR-corrected in the bilateral ROIs (Supplementary Figure 7). All participants consistently showed ipsilateral M1 deactivation, seven of nine participants showed ipsilateral PMd activation, and five of nine showed S1/Area 2 activation. We observed ipsilateral PMd activation and M1 deactivation as a group effect, essentially replicating the results in the YA group (Figure 2b top right).

**7 Causality analysis using the Linear Non-Gaussian Acyclic Model (LiNGAM)**

In the YA group, the stick rotation task activated the contralateral sensorimotor cortices and the ipsilateral PMd, S1 and Area 2, while the ipsilateral M1 remained deactivated (Figure 2b top right). In addition, during the stick rotation task but not the button press task, the ipsilateral PMd (especially the anterior part) consistently enhanced functional coupling with all contralateral seed regions (Figure 5). However, as these analyses do not address the causal relationship between brain activities, we used the LiNGAM to explore the causal relationship between brain activities across the eight bilateral ROIs (left or right PMd, M1, S1, or Area 2) during the stick rotation task in the YA group. This approach allowed us to explore the causal relationship (both positive and negative) between brain activities across multiple brain regions without requiring prior knowledge or specific hypotheses for the network structure (Ogawa et al., 2022). However, not all causal relationships obtained from this analysis can be clearly interpreted based on current neuroscientific knowledge. This approach will nevertheless enhance our understanding of the causal relationships among these cortical activities when younger adults perform the stick rotation task.

The LiNGAM (Shimizu et al., 2006) is a statistical method that addresses causal relationships among data. It estimates directed (either positive or negative) causal relationships within an unknown causal structure among variables based on observed data.

The LiNGAM is expressed by the following equation:

$x_{i}=\sum_{k\left( j \right)< k(i)} b_{ij}x_{j}+e_{i}\left( i=1,\ldots n \right),$ (1)

where the observed variable is$x_{i}$ expressed by the linear summation of other variables $x_{j} (j\neq i, j=1,\ldots n)$ and external influence $e_{i} (i=,\ldots,n)$. The causal ordering of the variables $x_{i}$is represented by $k(i)$. The external influence $e_{i}$ is assumed to be an independent and non-Gaussian distribution witha zeroo mean and nonzero variance. This assumption is crucial for determining the causal order among the variables. The connection weight $b_{ij}$ indicates a causal effect from observed variable $x_{j}$ to $x_{i}$. Equation 1 can be rewritten as

$\mathbf{x=Bx+e,}$ (2)

where $\mathbf{x}$ and $\mathbf{e}$ are vectors of $x_{i}$ and $e_{i}$, respectively. Since the LiNGAM assumes an acyclic graph structure, a causal order exists among the observed variables. Consequently, if rows and columns are rearranged accordingly, the $n\times n$ matrix $\mathbf{B}$, constituted by $b_{ij}$, will be lower triangular. Since many latent confounders (unobserved brain areas) might influence brain activities, in the present study we used the ParceLiNGAM (Tashiro et al., 2014) to estimate $b_{ij}$ and for causal ordering among brain activities. The ParceLiNGAM is more robust against latent confounders than the original LiNGAM (Tashiro et al., 2014) and we used an open-source library for LiNGAM (Ikeuchi et al., 2023).

**7.1 Data construction**

To apply the ParceLiNGAM to small-sample fMRI data, we used a method that aggregates data points across participants (Ogawa et al., 2022; Smith et al., 2011; Xu et al., 2014). After preprocessing the fMRI data as described in the main manuscript, we normalized and extracted time series data from the ROIs for each participant. To mitigate the impact of outliers, we used a robust scaling method, as follows:

$x_{\text{scale}}=\frac{x-Q_{2}}{Q_{3}-Q_{1}},$ (3)

where $Q_{2}$is the median of time series $x$, and $Q_{1}$and $Q_{3}$ represent first and third quantile of $x$, respectively. After normalization, we collected data points during the stick rotation task across all ROIs to construct a data matrix across all participants (Supplementary Figure 8).

**7.2 Statistic of the causal relationship**

We assessed the statistical significance of the causal effects in the estimated causal relationships using the bootstrap method. Based on previous studies (Ogawa et al., 2022; Shimizu et al., 2011; Xu et al., 2014), we constructed 1,000 distinct data matrices for bootstrap analysis in each group. Each matrix consisted of 25 randomly selected participants for each group. We then conducted the Wald test to evaluate the statistical significance of the causal effects between ROIs. Moreover, we confirmed whether the external influence $\mathbf{e=}\left( \mathbf{I-B} \right)\mathbf{x}$ satisfied the assumption of non-Gaussianity—one of the assumptions of the LiNGAM—using the Kolmogorov–Smirnov test (p < 0.05).

**7.3 Results and discussion**

Supplementary Figure 9 shows the causal relationship (order) among the eight ROIs during the stick rotation task in the YA group, as revealed by ParceLiNGAM. Although we cannot fully interpret all causal relationships obtained from this analysis, based on the neuroscientific knowledge available to date, part of the results can be interpreted in accordance with current neuroscientific knowledge.

During the stick rotation task, the contralateral (left) M1—the executive locus for motor control of the right hand—exerted a strong positive influence on the contralateral PMd. The contralateral PMd exerted a positive influence on multiple regions—including the ipsilateral PMd. Although the ipsilateral PMd enhanced functional coupling with all contralateral sensorimotor cortices (Figure 5), it received positive influences from the contralateral PMd and—less strongly—the M1. Since such an interhemispheric PMd–PMd interaction plays a very important role when the brain compensates for grasping for a damaged contralateral motor pathway during the recovery phase after unilateral spinal cord injury in non-human primates (Chao et al., 2019), this result suggests that the interhemispheric PMd–PMd interaction is also relevant when healthy younger brains complement the control of dexterous finger movement (Figure 5).

The ipsilateral M1—suppressed during the stick rotation task (Figure 2b top right)—was ranked lowest in causal order among all eight ROIs. Within ipsilateral (right) sensorimotor cortices, the M1 received positive influences from the PMd and—less strongly—the S1. This suggests that the ipsilateral PMd could determine whether the ipsilateral M1 is recruited or not during the stick rotation task. However, in our data the ipsilateral M1 remained suppressed (Figure 2b top right), most probably due to inhibitory (negative) influences from the contralateral side (in this case, from Area 2).

Although previous studies have reported ipsilateral PMd and M1 activations during dexterous finger movements (Loibl et al., 2011; Uehara et al., 2012; Verstynen et al., 2005), there seems to be a hierarchical order in their recruitment: the PMd is recruited almost immediately , but whether the M1 is recruited or not appears to be determined by the interaction between the positive influence from the ipsilateral PMd and the negative influence from the contralateral sensorimotor cortices.

The OA group, unlike the YA group, showed no significant causal relationships among ROIs. This could be related to excessive bilateral sensorimotor activity during the stick rotation task in the OA group (Figure 2b bottom right). Recently, using a spiking neural network model, we showed that excessive activity caused by a weakened inhibitory effect within a region in a network resulted in disrupted information transmission within the network (Park et al., 2023). We assume that excessive bilateral sensorimotor activities during the stick rotation task in the OA group (Figure 2b bottom right) might have disrupted information transmission in the brain network such that we observed no clear causal relationships among the sensorimotor cortices.

**References**

Chao, Z. C., Sawada, M., Isa, T., & Nishimura, Y. (2019). Dynamic reorganization of motor networks during recovery from partial spinal cord injury in monkeys. *Cerebral Cortex*, *29*(7), 3059–3073. https://doi.org/10.1093/cercor/bhy172

Ikeuchi, T., Ide, M., Zeng, Y., & Maeda, T. N. (2023). Python package for causal discovery based on LiNGAM. *Journal of Machine Learning Research: JMLR*, *24*(14), 1–8. https://github.com/jakobrunge/tigramite

Loibl, M., Beutling, W., Kaza, E., & Lotze, M. (2011). Non-effective increase of fMRI-activation for motor performance in elder individuals. *Behavioural Brain Research*, *223*(2), 280–286. https://doi.org/10.1016/j.bbr.2011.04.040

Moeller, S., Yacoub, E., Olman, C. A., Auerbach, E., Strupp, J., Harel, N., & Uǧurbil, K. (2010). Multiband multislice GE-EPI at 7 tesla, with 16-fold acceleration using partial parallel imaging with application to high spatial and temporal whole-brain fMRI. *Magnetic Resonance in Medicine*, *63*(5), 1144–1153. https://doi.org/10.1002/mrm.22361

Morita, T., Takemura, H., & Naito, E. (2023). Functional and structural properties of interhemispheric interaction between bilateral precentral hand motor regions in a top wheelchair racing Paralympian. *Brain Sciences*, *13*(5), 715. https://doi.org/10.3390/brainsci13050715

Ogawa, T., Shimobayashi, H., Hirayama, J. I., & Kawanabe, M. (2022). Asymmetric directed functional connectivity within the frontoparietal motor network during motor imagery and execution. *NeuroImage*, *247*, 118794. https://doi.org/10.1016/j.neuroimage.2021.118794

Park, J., Kawai, Y., & Asada, M. (2023). Spike timing-dependent plasticity under imbalanced excitation and inhibition reduces the complexity of neural activity. *Frontiers in Computational Neuroscience*, *17*, 1169288. https://doi.org/10.3389/fncom.2023.1169288

Shimizu, S., Hoyer, P. O., Hyvärinen, A., & Kerminen, A. (2006). A linear non-gaussian acyclic model for causal discovery. *Journal of Machine Learning Research: JMLR*, *7*(10), 2003–2030.

Shimizu, S., Inazumi, T., Sogawa, Y., Hyvärinen, A., Kawahara, Y., Washio, T., Hoyer, P. O., Bollen, K., & Hoyer, P. (2011). DirectLiNGAM: A direct method for learning a linear non-Gaussian structural equation model. *Journal of Machine Learning Research: JMLR*, *12*, 1225–1248.

Smith, S. M., Miller, K. L., Salimi-Khorshidi, G., Webster, M., Beckmann, C. F., Nichols, T. E., Ramsey, J. D., & Woolrich, M. W. (2011). Network modelling methods for FMRI. *NeuroImage*, *54*(2), 875–891. https://doi.org/10.1016/j.neuroimage.2010.08.063

Tashiro, T., Shimizu, S., Hyvärinen, A., & Washio, T. (2014). ParceLiNGAM: A causal ordering method robust against latent confounders. *Neural Computation*, *26*(1), 57–83. https://doi.org/10.1162/NECO_a_00533

Uehara, S., Nambu, I., Matsumura, M., Kakei, S., & Naito, E. (2012). Prior somatic stimulation improves performance of acquired motor skill by facilitating functional connectivity in cortico-subcortical motor circuits. *Journal of Behavioral and Brain Science*, *2*(3), 343–356. https://doi.org/10.4236/jbbs.2012.23039

Verstynen, T., Diedrichsen, J., Albert, N., Aparicio, P., & Ivry, R. B. (2005). Ipsilateral motor cortex activity during unimanual hand movements relates to task complexity. *Journal of Neurophysiology*, *93*(3), 1209–1222. https://doi.org/10.1152/jn.00720.2004

Xu, L., Fan, T., Wu, X., Chen, K. W., Guo, X., Zhang, J., & Yao, L. (2014). A pooling-LiNGAM algorithm for effective connectivity analysis of fMRI data. *Frontiers in Computational Neuroscience*, *8*, 125. https://doi.org/10.3389/fncom.2014.00125

Naito, E., Morita, T., Kimura, N., & Asada, M. (2021). Existence of interhemispheric inhibition between foot sections of human primary motor cortices: Evidence from negative blood oxygenation-level dependent signal. *Brain Sciences*, *11*(8), 1099. https://doi.org/10.3390/brainsci11081099

Morita, T., Asada, M., & Naito, E. (2019). Developmental changes in task-induced brain deactivation in humans revealed by a motor task. *Developmental Neurobiology*, *79*(6), 536–558. https://doi.org/10.1002/dneu.22701

Morita, T., Asada, M., & Naito, E. (2021). Examination of the development and aging of brain deactivation using a unimanual motor task. *Advanced Robotics*, *35*(13-14), 842–857. https://doi.org/10.1080/01691864.2021.1886168

Zeharia, N., Hertz, U., Flash, T., & Amedi, A. (2012). Negative blood oxygenation level dependent homunculus and somatotopic information in primary motor cortex and supplementary motor area. *Proceedings of the National Academy of Sciences of the United States of America*, *109*(45), 18565–18570. https://doi.org/10.1073/pnas.1119125109

**Supplementary Figures**


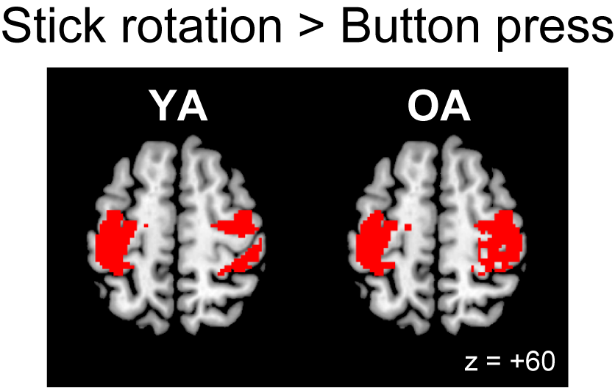


**Supplementary Figure 1. Brain regions more activated during the stick rotation task than during the button press task within the contralateral or ipsilateral ROIs in each group**

In the YA group, the stick rotation task activated all areas in the contralateral ROIs and the PMd, S1, and Area 2 in the ipsilateral ROIs more strongly than the button press task. In the OA group, the stick rotation task activated all areas in bilateral ROIs more strongly than the button press task. Activations are superimposed on a transverse section (z = +60) of the MNI standard brain. Abbreviations: MNI, Montreal Neurological Institute; YA, younger adult; OA, older adult.

**
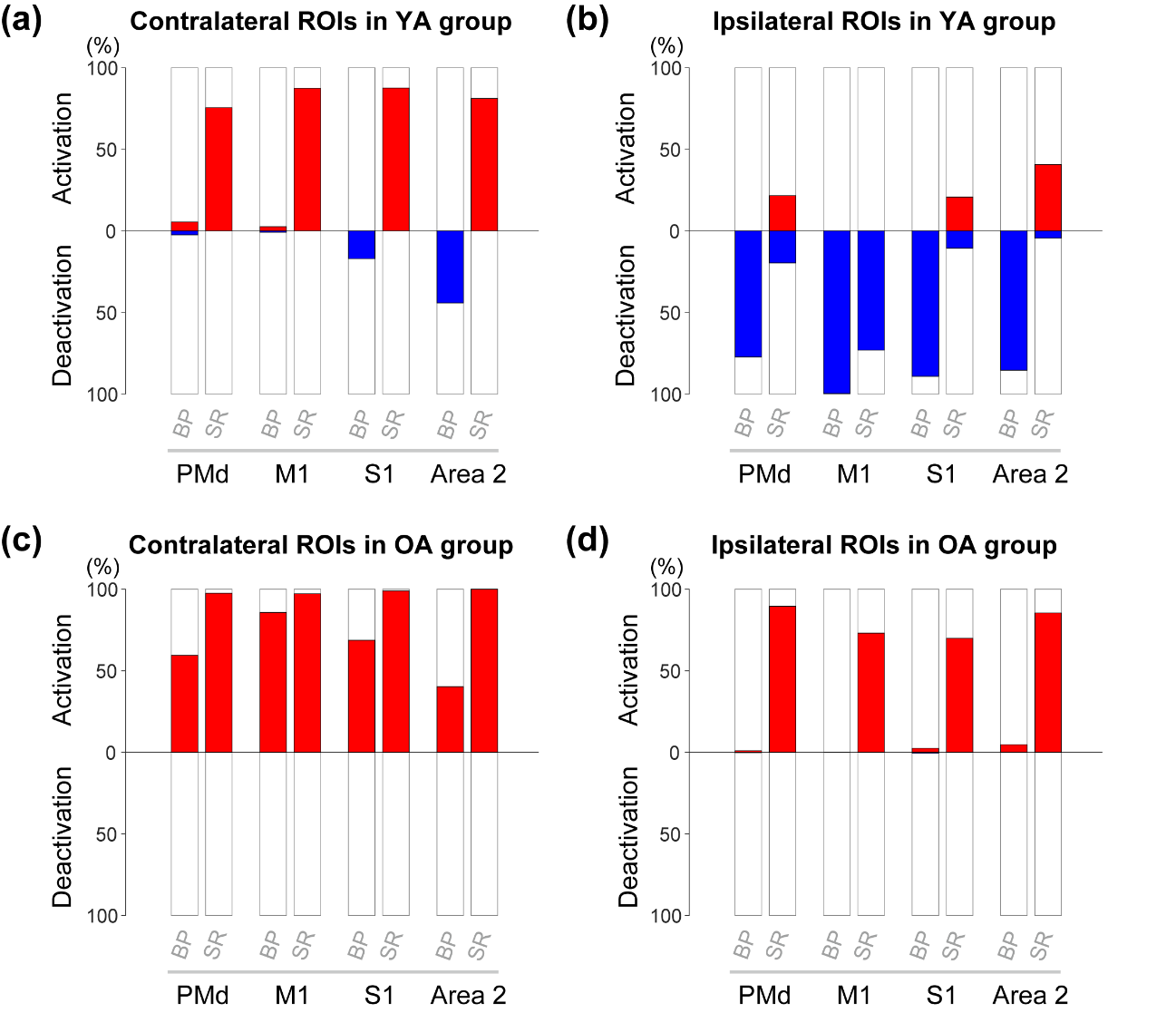
Supplementary Figure 2. Percentage of activated and deactivated voxels in each region-of-interest (ROI).** Results from contralateral (a) and ipsilateral (b) ROIs in the YA group. Results from contralateral (c) and ipsilateral (d) ROIs in the OA group. Red and blue bars indicate the percentage of activated and deactivated voxels, respectively. Overall, the results matched well with those obtained in the contrast analysis (Figure 2b). Abbreviations: YA, younger adult; OA, older adult; BP, button press task; SR, stick rotation task.


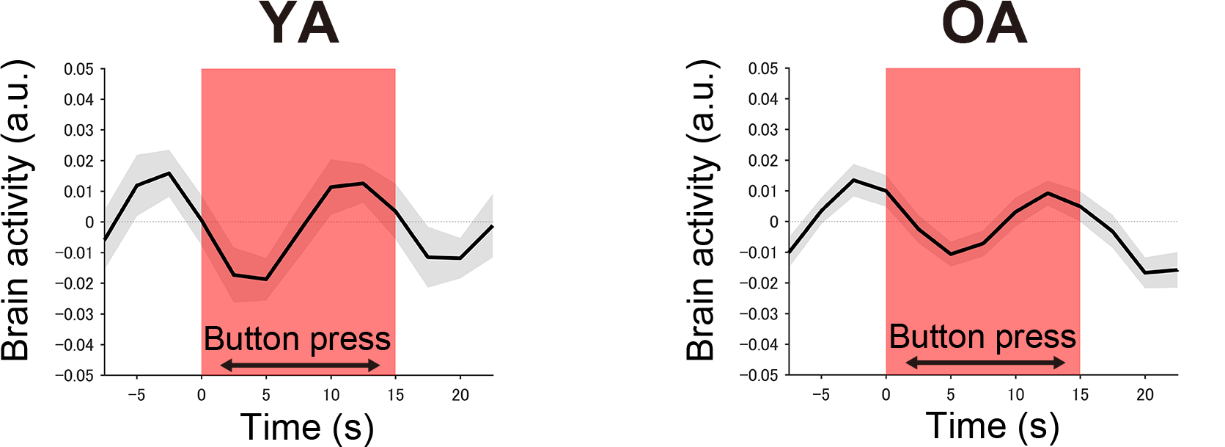


**Supplementary Figure 3. Temporal profile of brain activity during the button press task in the YA and OA groups.** We extracted the time-course data from a sphere with a 4 mm radius around the peak of the M1 deactivation ([40, -22, 66]) during the button press task in the YA group. Left, YA group; right, OA group. The red period indicates the button press task epoch. The x-axis shows the time course where the start of the task epoch is set to 0 s. The y-axis indicates the brain activity level. Gray shaded regions in the graph indicate the SEM.


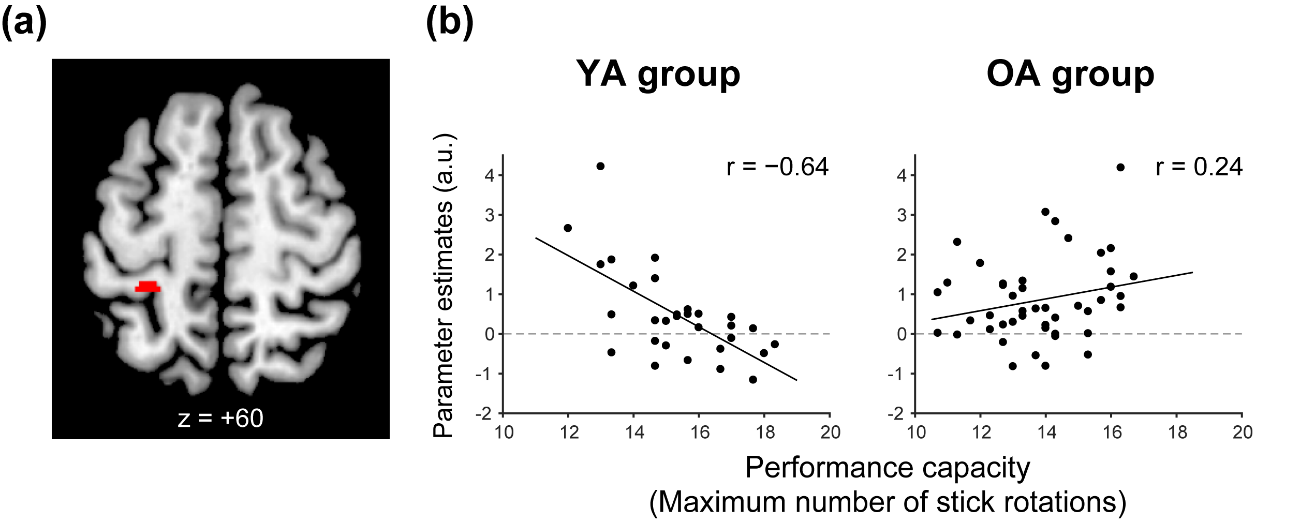


**Supplementary Figure 4. Brain region where activity is negatively correlated with performance capacity in the contralateral ROIs in the YA group** (a) Activity in the contralateral S1/Area 2 negatively correlated with the performance capacity of stick rotation in the YA group. We superimposed the activity on the horizontal section of z = +60 of the MNI standard brain. (b, c) Interparticipant correlation between performance capacity (x-axis) and brain activity of the significant S1/Area2 cluster (y-axis) in the YA (b) and OA (c) groups. Solid lines in each panel indicate linear regression lines fitted to the data. Abbreviations: MNI, Montreal Neurological Institute; YA, younger adult; OA, older adult; a. u., arbitrary unit.


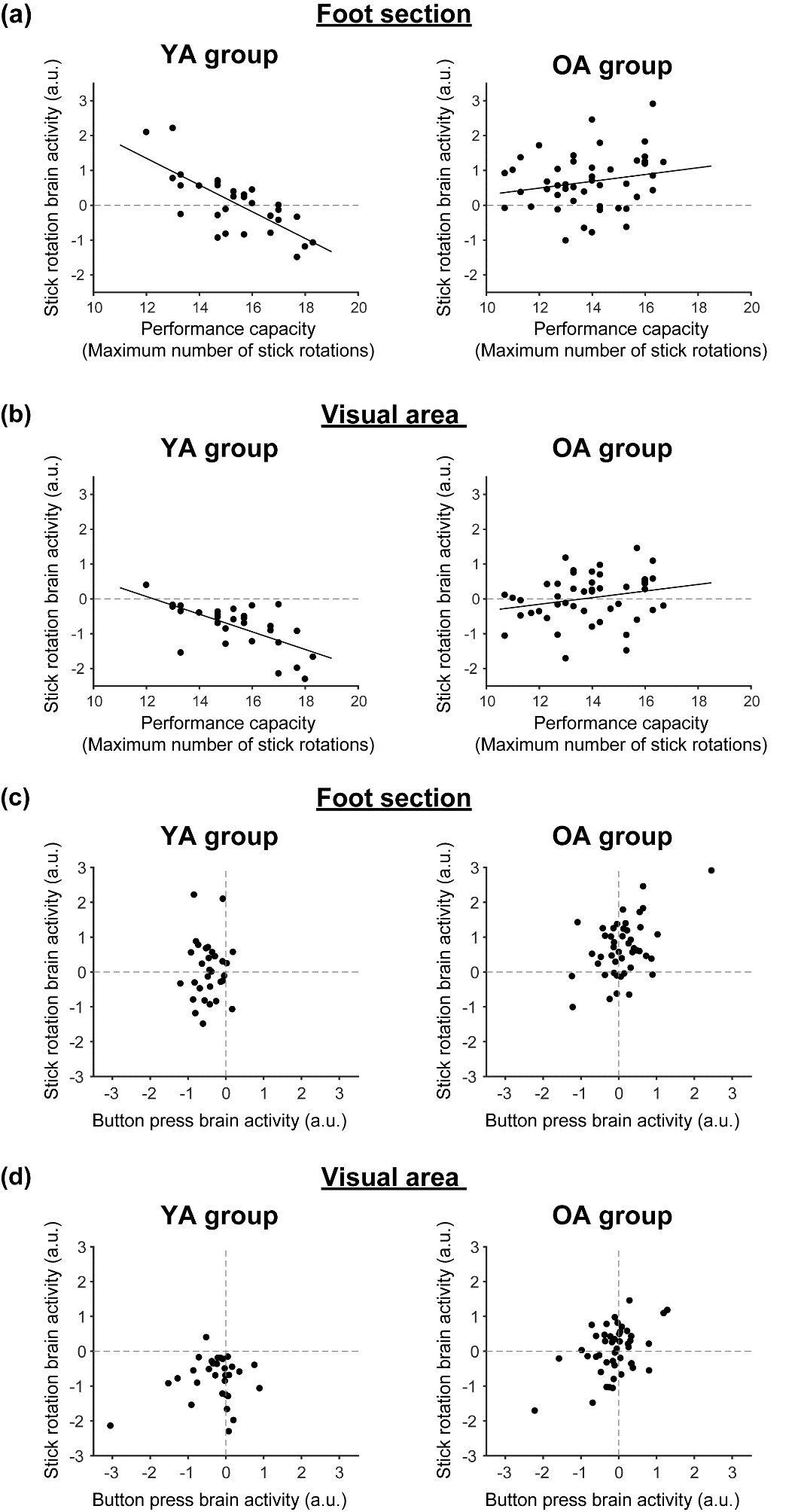


**Supplementary Figure 5. Other brain regions where activity is negatively correlated with performance capacity in the whole brain in the YA group** (a, b) Interparticipant correlation between performance capacity (x-axis) and brain activity (y-axis) of the foot section (a) and the visual area (b) in the YA (left panel) and OA (right panel) groups. (c, d) Relationship of brain activity in the foot section (c) and the visual area (d) between during the button press task (x-axis) and the stick rotation task (y-axis) in the YA (left panel) and OA (right panel) groups. Abbreviations: YA, younger adults; OA, older adults; a.u., arbitrary unit.


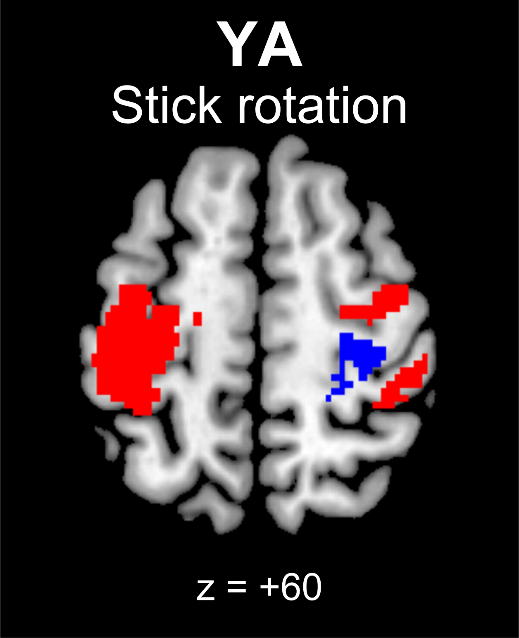


**Supplementary Figure 6. Ipsilateral PMd, S1, and Area 2 activation and M1 deactivation in 8-mm filtered functional images.** We observed ipsilateral PMd, S1, and Area 2 activation (red) and M1 deactivation (blue) in 8-mm filtered images. The results replicated the ipsilateral activation and deactivation patterns observed when analyzing the 4-mm filtered images (Figure 2b). We superimposed the activation and deactivation on the horizontal section of z = +60 of the MNI standard brain. Abbreviations: MNI, Montreal Neurological Institute.


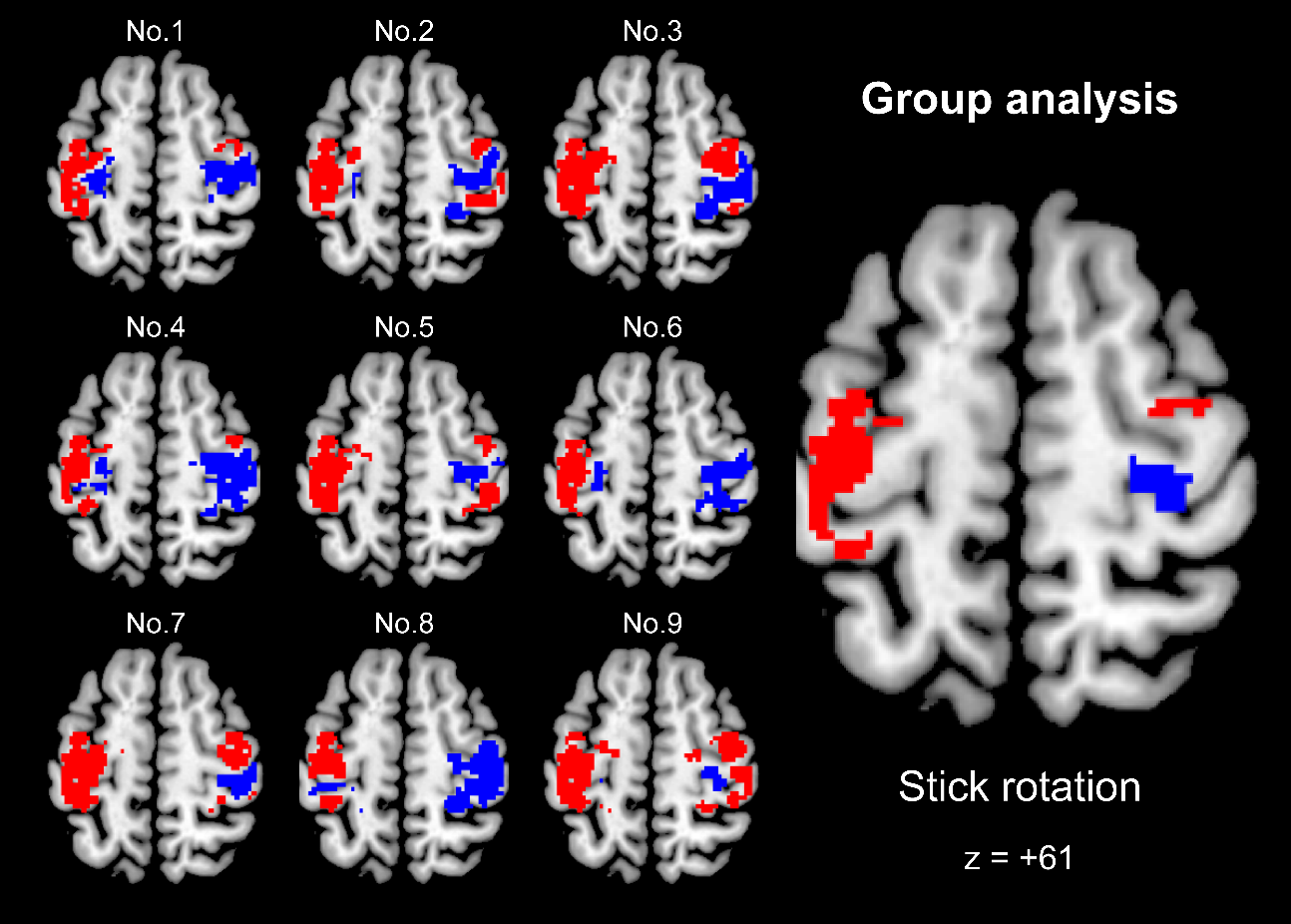


**Supplementary Figure 7. Individual brain activation and deactivation in nine other younger adults who performed the stick rotation task as well as their group effect.** We superimposed activation (red) and deactivation (blue) in individual participants and their group effect on the horizontal section of z = +61 of the MNI standard brain. Abbreviations: MNI, Montreal Neurological Institute.


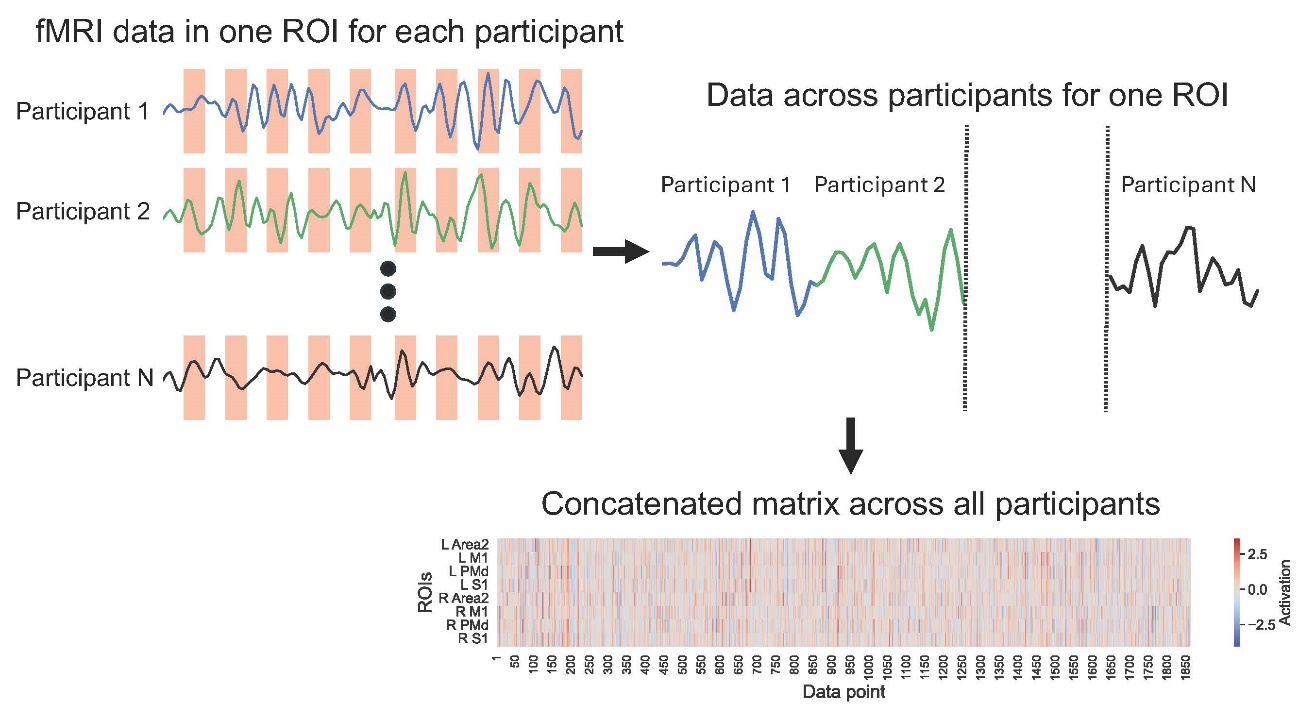


**Supplementary Figure 8. Data matrix using fMRI data.** Areas in red indicate data during each task epoch. We concatenated the data from each participant for each ROI to generate a data matrix.

**
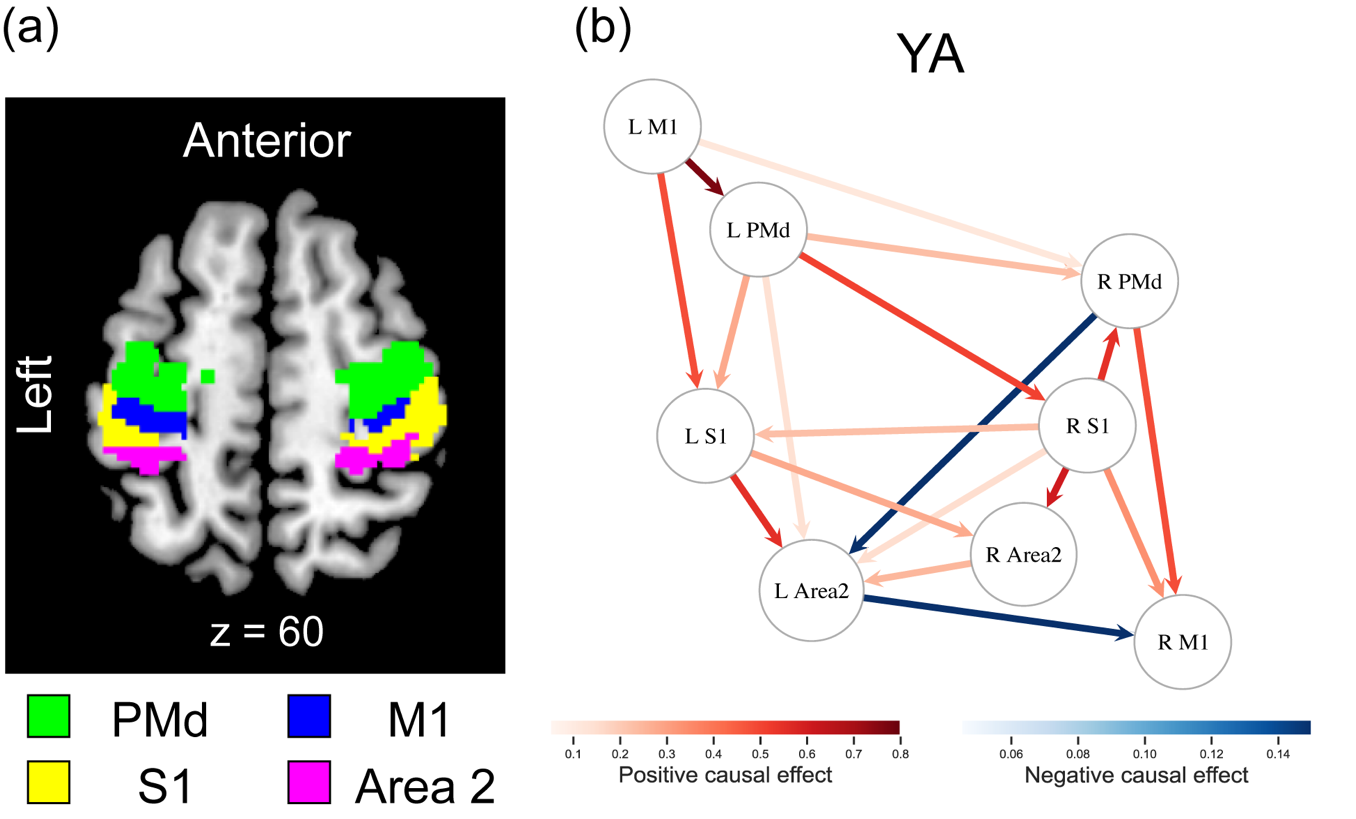
Supplementary Figure 9. Estimated causal influences (order) among the eight ROIs (a) during the stick rotation task in the YA group using ParceLiNGAM.** (a) Eight selected ROIs of the left and right PMd, M1, S1, and Area 2 superimposed on an horizontal section of z = +60 of the MNI standard brain (see also Figure 2a). (b) Significant causal relationships (order) among ROIs. Each circle represents a selected ROI. Red and blue arrows indicate positive and negative causal effects from one ROI to another, respectively. Causal effects are shown only for p *<* 0.05 through the Wald test. Abbreviations: MNI, Montreal Neurological Institute.

**Supplementary Table 1. Brain regions more activated during the stick rotation task than during the button press task within contralateral or ipsilateral ROIs in each group**

|  | Size | t-value | x | y | z |  | Anatomical identification |
| --- | --- | --- | --- | --- | --- | --- | --- |
| **YA group** | 1764 | 10.07 | −44 | −18 | 56 |  | Area 3b |
|  |  | 9.01 | −30 | −18 | 64 |  | Area 6d1 |
|  |  | 8.64 | −32 | −8 | 66 |  | PrG |
|  | 518 | 8.97 | 42 | −34 | 56 |  | Area 2 |
|  |  | 7.82 | 52 | −24 | 54 |  | Area 1 |
|  | 453 | 7.54 | 34 | −6 | 64 |  | PrG |
|  |  | 6.22 | 26 | −12 | 60 |  | Area 6d1 |
|  |  |  |  |  |  |  |  |
| **OA group** | 1583 | 8.07 | −46 | −24 | 60 |  | Area 1 |
|  |  | 7.27 | −38 | −30 | 50 |  | Area 4p |
|  |  | 7.25 | −44 | −34 | 58 |  | Area 3b |
|  | 1704 | 7.59 | 30 | −12 | 58 |  | Area 6d1 |
|  |  | 7.25 | 36 | −14 | 66 |  | PrG |
|  |  | 7.21 | 34 | −34 | 46 |  | Area 2 |

Height threshold, p < 0.005. uncorrected; extent threshold, p < 0.05, FWE corrected within the contralateral and ipsilateral ROIs, separately, using SVC. Size refers to the number of significant voxels. For the anatomical identification of peaks, we considered only cytoarchitectonic areas available in the anatomy toolbox with > 30% probability. We reported the cytoarchitectonic area with the highest probability for each peak. When cytoarchitectonic areas with >30% probability were unavailable, we simply provided the anatomical location of the peak. In each cluster, we report peaks separated by > 8 mm in the order of larger t-values. To facilitate visualization, we avoided reporting a peak for each cluster when identified in the cytoarchitectonic area or anatomical structure already reported for a peak with a higher t-value.

Abbreviations: PrG, precentral gyrus; SVC, small volume correction.
